# Functional characterization of the Serine acetyltransferase family genes uncovers the diversification and conservation of cysteine biosynthesis in tomato

**DOI:** 10.3389/fpls.2022.913856

**Published:** 2022-09-21

**Authors:** Danmei Liu, Min Li, Ting Guo, Juanjuan Lu, Yafang Xie, Yuan Hao, Longdan Wang, Dan Zhao, Liping Zhang, Zhiqiang Liu, Zhuping Jin, Yanxi Pei

**Affiliations:** ^1^School of Life Science, Shanxi University, Taiyuan, China; ^2^Shanxi Key Laboratory for Research and Development of Regional Plants, Taiyuan, China

**Keywords:** Serine acetyltransferase, tomato, enzymatic activity, subcellular localization, expression pattern, OE-SlSERAT1;1

## Abstract

Sulfur-containing compounds are essential for plant development and environmental adaptation, and closely related to the flavor and nutrition of the agricultural products. Cysteine, the first organic sulfur-containing molecule generated in plants, is the precursor for most of these active substances. Serine acetyltransferase (SERAT) catalyzes the rate-limiting step of its formation. However, despite their importance, systematic analyses of these enzymes in individual species, especially in economically important crops, are still limited. Here, The SERAT members (SlSERATs, four in total) were identified and characterized in tomato. Phylogenetically, the four SlSERAT proteins were classified into three subgroups with distinct genomic structures and subcellular localizations. On the function, it was interesting to find that SlSERAT3;1, possessed a high ability to catalyze the formation of OAS, even though it contained a long C-terminus. However, it retained the essential C-terminal Ile, which seems to be a characteristic feature of SERAT3 subfamily members in Solanaceae. Besides, SlSERAT1;1 and SlSERAT2;2 also had high activity levels and their catalyzing abilities were significantly improved by the addition of an OAS-(thiol)-lyase protein. At the transcriptional level, the four SlSERAT genes had distinct expression patterns during tomato plant development. Under abiotic stress conditions, the chloroplast-localized SlSERATs were the main responders, and the SlSERATs adopted different strategies to cope with osmotic, ion toxicity and other stresses. Finally, analyses in the loss-of-function and overexpression lines of SlSERAT1;1 suggested that function redundancy existed in the tomato SERAT members, and the tomato SERAT member was ideal target for S-assimilation manipulating in molecular breeding.

## Introduction

Sulfur is an essential macronutrient found in plants. Although sulfur is generally not a structural component of biomolecules, it is always involved in the catalytic or electrochemical functions of molecules in which it is a component (Leustek et al., 2000). Sulfur is taken up by plants in the form of sulfate and then reduced and assimilated into cysteine (Cys), the first stable organic sulfur-containing compound (Romero et al., 2014). Cys occupies a central position in plant metabolism, being not only an important amino acid in proteins, but also a donor molecule of reduced sulfur for the synthesis of many essential biomolecules and metabolites (Wirtz et al., 2001; Ba et al., 2021). Moreover, because plants are the major source of reduced sulfur in animal and human nutrition, Cys biosynthesis has attracted attentions from both biologists and agronomists (Hell et al., 2002).

The biosynthesis of Cys is mainly divided into two steps. First, with L-serine and acetyl-coenzyme A as the substrates, Serine acetyltransferase (SERAT) catalyzes the formation of *O*-acetylserine (OAS), which links serine metabolism to Cys biosynthesis (Liu et al., 2020). Subsequently, under the catalysis of OAS-(thiol)-lyase (OASTL), OAS condenses with sulfide to form Cys (Kawashima et al., 2005; Krueger et al., 2009; Tavares et al., 2015). In plants, the activity of OASTL is always 100–300 greater than that of SERAT, and OAS biosynthesis is the rate-limiting step in Cys biosynthesis (Noji et al., 1998; Krueger et al., 2009). The activity of SERAT is post-translationally regulated by the formation of the Cys synthase complex (CSC; Saito, 2000; Krueger et al., 2009; Tavares et al., 2015). The protein complex formed by SERAT and OASTL allows SERAT to gain full activity, while OASTL is conformationally changed and inactivated. The OAS generated by SERAT is able to dissociate the CSC, release the free-formed OASTL and promote the formation of Cys in a certain concentration range (Campanini et al., 2005; Haas et al., 2008).

At the protein structure level, each SERAT monomer consists of an N-terminal *α*-helical domain and a C-terminal left-handed parallel *β*-helix domain, which is also a characteristic of the acyltransferases (Wirtz et al., 2001; Jez and Dey, 2013; Kumar et al., 2014). There are two protein-interaction domains in the SERAT protein sequence, a central homomerization domain for SERAT–SERAT interactions and a C-terminal heteromerization domain for SERAT-OASTL interactions (Wirtz et al., 2001), in which the C-terminal Ile is critical for molecular recognition by OASTL proteins (Francois et al., 2006). In addition, the C terminus, which is highly conserved among SERAT members, is also the active site and the substrate-binding site (Wirtz et al., 2001; Jez and Dey, 2013).

As a rate-limiting enzyme during Cys biosynthesis, SERATs participate in many developmental processes, as well as in plant environmental adaptation. Studies in *Arabidopsis* showed that the OAS formed by SERAT is indispensable for plant viability. The Arabidopsis quintuple SERAT mutant is embryo-lethal, and three out of the five quadruple mutants produce dwarf phenotypes. However, none of the five single mutants of Arabidopsis SERATs show visible phenotypic changes under normal growth or stress conditions, indicating that function redundancy exists among SERAT isoforms, though the contribution to the cellular OAS is different for each SERAT member (Watanabe et al., 2008). On the other hand, because Cys participates in the biogenesis of many sulfur-containing compounds in food and feed, agronomists have focused on the manipulation of SERAT levels in different plant materials to improve plant nutritional values. The overexpression of Arabidopsis SERAT in tobacco (*Nicotiana tabacum* L.) improves Cys, Met and glutathione production in leaves, while the maternal overexpression of SERAT in vegetative tissues leads to the accumulation of high-Met zein in maize (*Zea mays* L.; Wirtz and Hell, 2007; Xiang et al., 2018). However, when an Arabidopsis SERAT is overexpressed in lupin (*Lupinus angustifolius* L.), no significant alteration in the total Cys and Met concentrations in the mature seeds occur, indicating that improving the nutritional quality of different crops by controlling SERAT expression is feasible, but having specificity in each plant species (Tabe et al., 2010). SERATs also participate in the plant resistance to various stresses. In T. goesingense (*Thlaspi goesingense*), the insensitivity of its SERAT to Cys plays an important role in its resistance to hyper accumulated nickel (Na and Salt, 2011). In *Arabidopsis*, the expression of SERAT4 is greatly induced under cadmium stress and sulfur deficiency (Kawashima et al., 2005), and the exogenous expression of the T. goesingense SERAT in *Arabidopsis* improves its tolerance to multiple heavy metals (Freeman and Salt, 2007). Under drought-stress conditions, SERAT expression is inhibited in the leaves, impeding the growth of the aboveground tissues, which reduces plant consumption and helps prolong plant lives (Ahmad et al., 2016).

In this study, four SERAT genes were cloned and characterized from the economically important crop tomato (Wang et al., 2018). Their potential functions during plant development and environmental adaption were also explored, and especially, the function of SlSERAT1;1 was investigated *in vivo* through genetic means. This work will help widen our understanding of the S-assimilation system in higher plants, and provide fundamental support for relative biotechnical manipulating in tomato.

## Materials and methods

### Plant material, growth conditions and treatments

Tomato (*Solanum lycopersicum* L., cv. MicroTom) seeds were first surface sterilized sequentially with 95% ethanol for 2 min and 20% bleach for 20 min, washed with double-distilled water, and placed on three layers of wet filter papers in a petri dish to geminate.

For abiotic stress treatments, 7-day-old seedlings (after germination) were used. For Salt and heavy metal (Cd^2+^) treatment, the 7-day-old seedlings were placed in new petri dishes, in which the filter papers were wet with 8 ml NaCl (250 mM) or CdCl_2_ (5 mM) solution. For draught treatment, the 7-day-old seedlings were placed on dry filter papers. Seedlings in all the above groups were sampled at 0, 3, 6, 9, 12, and 24 h after treatment, then quick-frozen in liquid nitrogen and stored at −80°C for further analysis (Fang et al., 2014).

For sulfur-deficit treatments, the 7-day-old seedlings were divided into two groups, one group was grown in a Murashige and Skoog (MS) solution with full sulfate (+S, 1.7 mM), while the other group was grown in a modified MS solution (−S, 0.1 mM) in which most of its sulfate salts were substituted with chloride salts. The treated seedlings were then sampled at 1, 3, 5 and 7 days of growth, and the materials were collected and stored (Tavares et al., 2015).

For the other tomato materials used in this work, when the hypocotyls of these plant seedlings reached 2 cm, all the seedlings were transferred into the soil and cultivated in a growth chamber under 24°C/16-h day and 18°C/8-h night conditions.

### Gene cloning and sequence analyses

To obtain the gene sequences encoding tomato SERATs, the five Arabidopsis SERAT protein sequences were used as queries for the tblastn search of the tomato cDNA database. The amplification primers were designed using the Primer Premier 5 software (Singh et al., 1998), and the primer sequences are listed in Supplementary Table S1. Total RNA was extracted from a mixture of tomato tissues, and cDNA was synthesized using the First-Strand cDNA Synthesis Kit in accordance with the manufacturer’s instructions (ABM, Nanjing, China). The PCR amplification was performed as described previously (Liu et al., 2019), with the annealing temperature set at 55°C and the extension time set at 90 s. The purified PCR products were then cloned independently into the pMD-18 T vector (TaKaRa, Kyoto, Japan) and sequenced to confirm the coding domain sequence (CDS) of each gene.

The gene structures were constructed based on the alignment results between the CDS and the genome sequence of each gene. The ClustalX (Thompson et al., 2002) and Gendoc software packages were used to conduct a multiple protein sequence alignment. For the phylogenetic analysis, MEGA 7.0 (Kumar et al., 2016) software was used, and an Arabidopsis OASTL family protein, OAS-A1, was used as an outgroup. The cis-acting regulatory elements were analyzed using the PlantCARE website^1^ (Lescot et al., 2002) with 3,000 bp upstream sequences of every *SlSERAT* gene.

### Subcellular localization

For transient transformations, CDSs of all the tomato SERAT genes were cloned independently into the vector p1305-GPF, which possesses a 35S promoter and a C-terminal GFP tag. Then, the generated constructs were transformed into the *Agrobacterium tumefaciens* strain EHA105 separately before being infiltrated into tobacco (*Nicotiana benthamiana*) leaves as described previously (Liu et al., 2019). For fluorescence signal detection, a confocal laser scanning microscope (ZEISS, Germany) was used, and the excitation/emission spectra were 488/493–598 for GFP, 633/647–721 for chlorophyll auto-fluorescence and 561/595–670 for RFP. For mitochondrial localization, mt-rk was used as a control (Nelson et al., 2007). The auto-fluorescence of chlorophyll was used to indicate the chloroplast positions. A peroxisome marker, px-rk was used as an indicator of the peroxisome (Nelson et al., 2007).

### Protein purification and enzymatic activity assay

The mature coding regions of tomato *SERAT* genes were first cloned independently into the pCold vector for the exogenous expression of the recombinant proteins having an N-terminal His-tag. After inducing the expression of these tomato SERAT proteins in *Escherichia coli* strain BL21 with IPTG, the recombinant proteins were purified using a Ni-NTA Sefinose™ resin (Sangon Biotech, Shanghai, China) and detected with SDS-PAGE to determine their purity. Then, the activities of these exogenously expressed and purified recombinant proteins were analyzed.

The activities of these tomato SERATs were determined as described previously with minor modifications (Harms et al., 2000). Briefly, the purified recombinant proteins were first added into a reaction mixture containing 50 mM Tris-HCl (pH 7.6), 1 mM EDTA, 20 mM L-serine and 0.1 mM acetyl-CoA. Then, the reaction was incubated at 30°C for 30 min. The generated CoA was detected by adding 50 μl of 1 mM 5,5′-dithiobis-(2-nitrobenzoic acid), and the yellow nitrobenzoic acid produced was monitored at 412 nm. A standard curve was constructed with control solutions containing all the compounds and different concentrations of CoA (0–200 μM).

To analyze the effects of the OASTL protein on the activities of these tomato SERAT proteins, the SlOAS6 protein was exogenously expressed and purified as described previously (Liu et al., 2019). And this time, the CDS of *SlOAS6* was cloned into the pET28a vector for prokaryotic expression. Then, the SERAT activities were compared with, or without, the SlOAS6 protein.

### Expression analysis by real-time PCR

For gene expression analysis, total RNA was extracted from different tomato tissues (red fruit, yellow fruit, breaker fruit, immature green fruit, pedicel, carpel, stamen, petal, sepal, inflorescence, old leaf, young leaf, stem, seedling, and root), as well as the stress-treated seedling materials, and transcribed into first-strand cDNA as described above. Then, the qPCR reactions were performed on a 7,500 Fast Real-Time PCR System (Applied Biosystems, United States), and the relative expression levels of each gene were calculated as described previously (Livak and Schmittgen, 2001). The *SAND* gene (SGN-U316474) was used as an internal control for the gene expression pattern analysis, while the *ACTIN* gene (SGN-U581238) was used to normalize the results of the gene expression changes under different treatment conditions (Exposito-Rodriguez et al., 2008). All the primers used in this analysis were listed in Supplementary Table S1.

### Chloroplast extraction and SERAT activity assays In tomato seedlings under salt stress treatment

Salt stress treatment were performed as described above, and the salt-treated seedlings were obtained at 0, 6, 12, and 24 h for further analyzed. SERAT activities were analyzed with proteins extracted from both the whole cell and the isolated chloroplasts. For total SERAT activity assay, total soluble proteins were first extracted from the seedlings (100 mg) with the phosphate buffered solution (0.05 M, pH 7.0, 1 ml). For chloroplast SERAT activity assay, the chloroplast proteins were first extracted using an chloroplast-protein extraction kit (BioRab Technology, Beijing, China) with 500 mg treated tissues (from about 60 seedlings for each replicate). Briefly, the chloroplasts were isolated using a method based on differential centrifugation according to the manufacturer’s instructions, and the chloroplast proteins were obtained with a nonenzymatic chloroplast dissociation solution. After all the proteins get prepared, the enzyme activity was analyzed as described above.

### Construct development and tomato transformation

To generate the loss-of-function plant material for *SlSERAT1;1*, the CRISPR/Cas9 binary vector pKI1.1R was used (Tsutsui and Higashiyama, 2017). Two *SlSERAT1;1* target sites (gRNA1 and gRNA2) were designed and evaluated on the CRISPRdirect^2^ and the RNAstructure websites.^3^ Then the 20-bp oligos of the gRNAs were inserted into the pKI1.1R vector. Meanwhile, SlSERAT1;1-overexpression (OE-SlSERAT1;1) constructs were generated using a modified pCAMBIA2300 vector (with a 35S promoter and a 3 × FLAG tag before the MCS site) with the mature coding region of *SlSERAT1;1*. The resulting constructs were further transformed into the tomato cv. MicroTom through *A. tumefaciens* (strain EHA105) mediated transformation as described before (Liu et al., 2014). For gene-editing analysis of these transformed plants, fragments flanking the gRNA targeting sequences were amplified from the genomic DNA and sequenced. The primers used for vector construction and mutation analyses were listed in Supplementary Table S1.

### Cys content assays in plants

For Cys content assay, the leaf tissues (100 mg) from both the wide type and the transgenic tomato plants were first homogenized with 5% (v/v) perchloric acid solution (0.4 ml), reacted with the acid ninhydrin for 10 min at 100°C, and then the absorbance of the reaction was measured at 560 nm to determine the Cys concentration as reported previously (Fang et al., 2016; Liu et al., 2019).

### Statistical analysis

All the experiments were conducted with three biological and three technical replicates. The data were presented as means ± standard errors (SEs). The significance analyses were performed using SPSS 19.0 software (IBM SPSS, Chicago, IL, United States), with different letters (*p* < 0.05), ^*^(*p* < 0.05) and ^**^(*p* < 0.01) indicating statistically significant effects.

## Results

### Cloning and identification of the SERATs in tomato

To obtain the CDSs of *SERATs* in tomato, the protein sequences of Arabidopsis *SERATs* (all five members) were used as queries in the tblastn search of the tomato cDNA database, and four putative SERAT-encoding genes that displayed high similarity levels (*E*-value < e^−10^) were found. Then, the CDSs of these four tomato *SERAT* genes were PCR amplified, cloned, and sequence confirmed.

**Figure 1:**
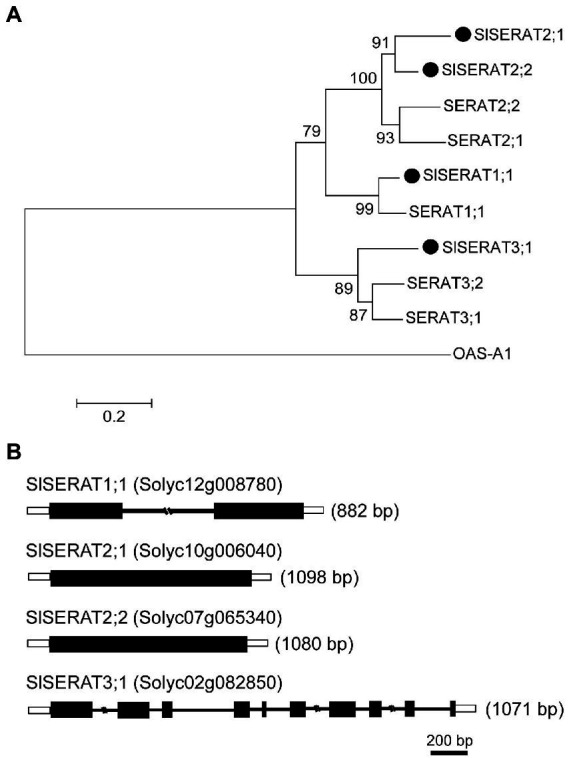
Phylogenetic relationship and the genomic organization of the Serine acetyltransferase (SERAT) family members in tomato. **(A)** The neighbor-joining tree of SERAT proteins from Arabidopsis (labeled as SERATs) and tomato (labeled as SlSERATs). The Arabidopsis OASTL family protein OAS-A1 was used as an outgroup control. **(B)** Gene structures of the tomato SERATs (SlSERATs). The numbers in brackets represent the lengths of the CDSs of the genes.

**Figure 2:**
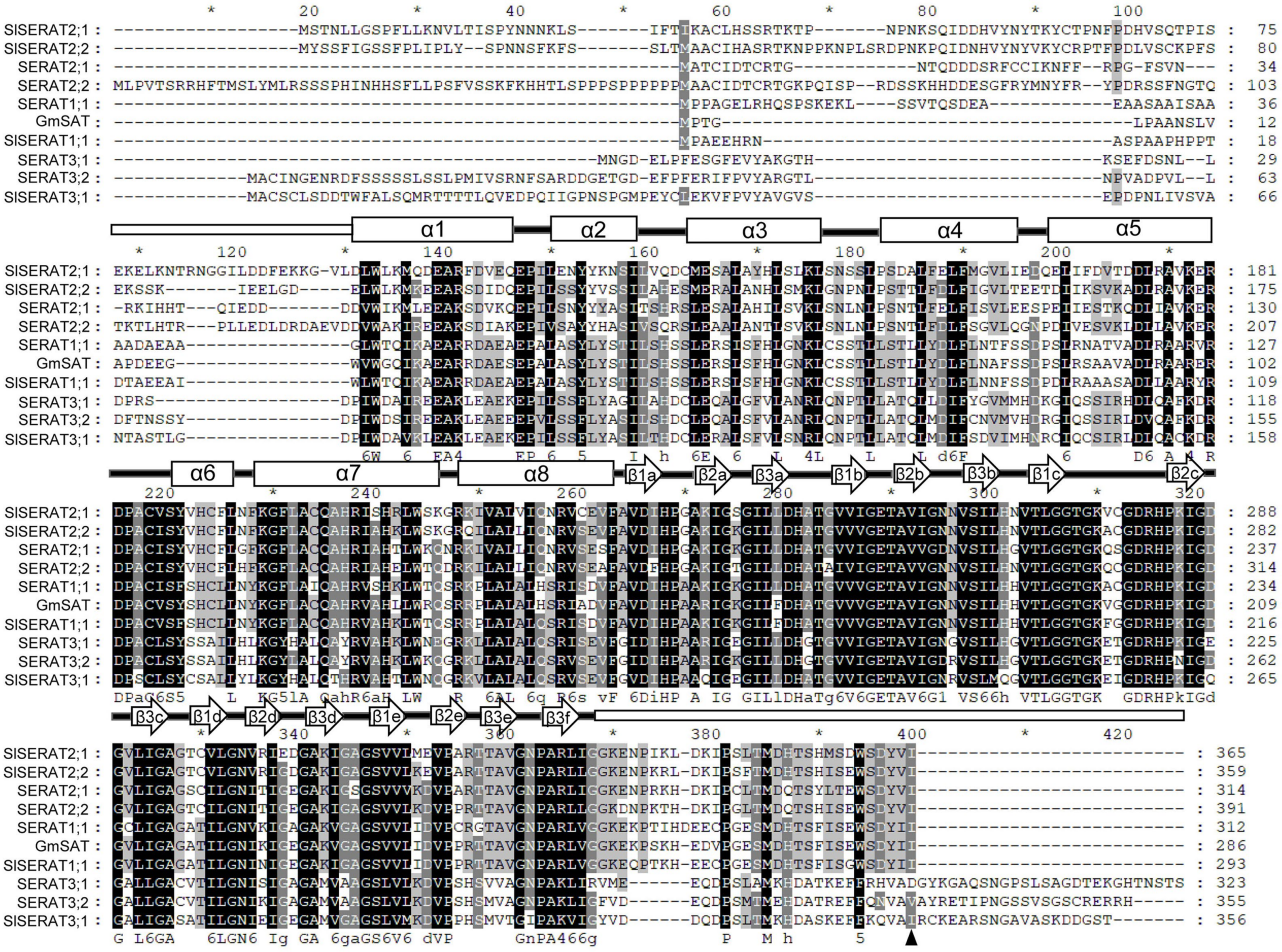
Comparison of the SERAT protein sequences from tomato, Arabidopsis and soybean. The secondary structures are shown above the sequences: white rectangles, *α*-helix; white arrows, *β*-sheets; and black bars, loops. The essential C-terminal Ile is indicated by a black triangle. White letters on a black background indicate the most conserved amino acids (identical in all the analyzed protein sequences), and the fading background color indicates a reduction in the conservation.

Phylogenetically, these four tomato SERATs were classified into three subclades, and have thus been named as SlSERAT1;1, SlSERAT2;1, SlSERAT2;2 and SlSERAT3;1 (Figure 1A). In accordance with a previous report (Watanabe et al., 2008), tomato SERATs belonging to different subclasses had unique exon-intron structures. Thus, *SlSERAT1;1* had one intron, *SlSERAT2;1* and *SlSERAT2;2* had no introns, while *SlSERAT3;1* had 10 exons and nine introns (Figure 1B). Moreover, the protein sequences of these SlSERATs were also compared with the SERAT proteins from *Arabidopsis* and soybean. The multiple protein sequence alignment showed that the N-terminal *α*-helix and C-terminal *β*-sheet were conserved in the SlSERATs. SlSERAT3;1 had an extended C-terminus as its homologs in *Arabidopsis*, but the essential Ile was still retained in its protein sequence, which differed from its Arabidopsis homologs in the same subgroup (Figure 2). Moreover, it is interesting to find that this amino acid site (Ile) was highly conserved among species from the Solanaceae family (Supplementary Figure S1).

### Subcellular localization of the SlSERAT proteins

**Figure 3:**
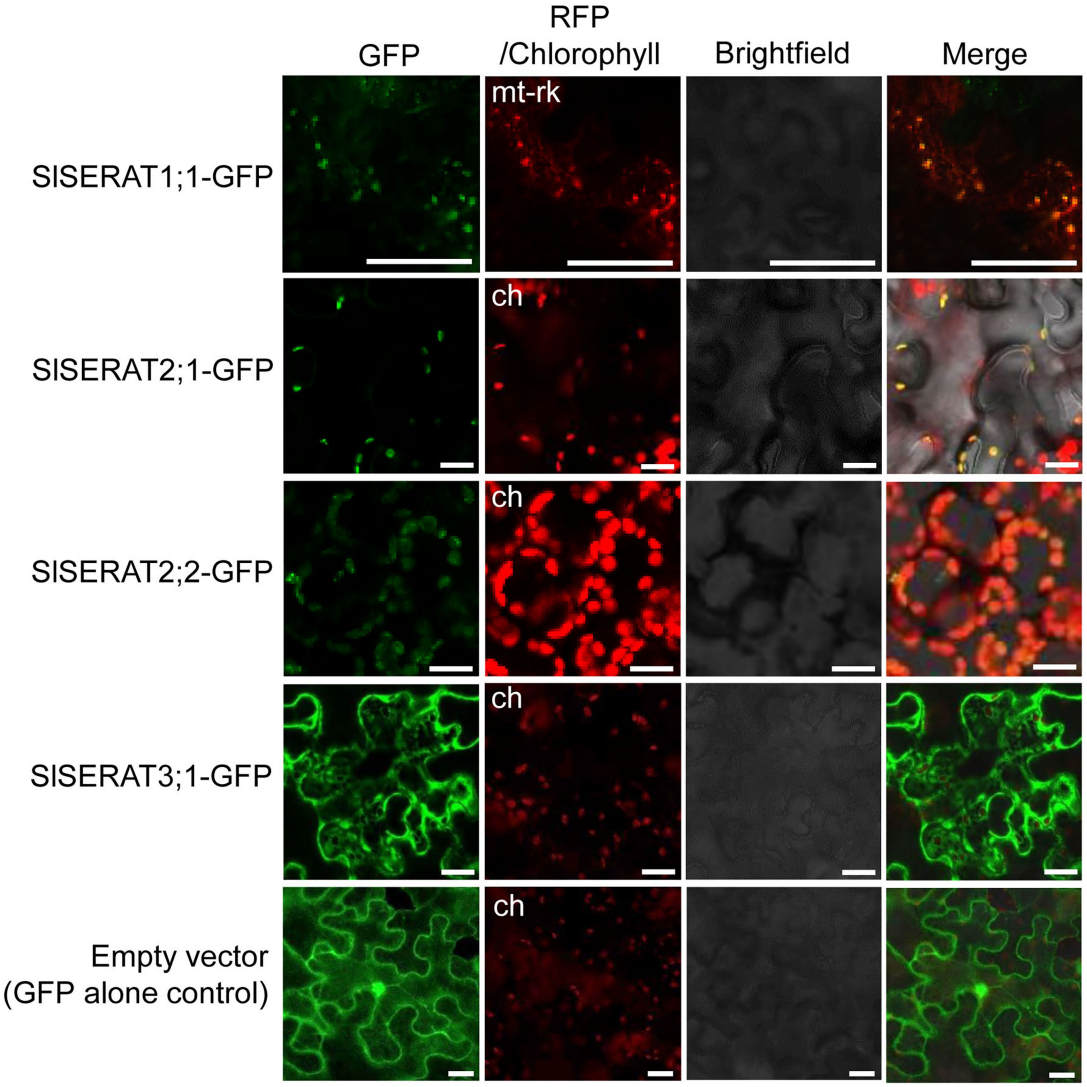
Subcellular localization of the tomato SERAT proteins. For SlSERAT1;1, the mitochondrial marker (mt-rk, RFP fusion) was used to indicate the position of mitochondria. For SlSERAT2;1 and SlSERAT2;2, the autofluorescence of the chloroplasts (ch) corroborated their chloroplast localization. The signals of SlSERAT3;1 spread all over the cytoplasm. GFP in the empty vector was expressed all over the cell, and the signals could be spotted in the plasma membrane, the cytoplasm, and the nucleus.

The subcellular localizations of these tomato SERATs were determined in tobacco leaf epidermal cells using proteins fused with GFP at the C-termini. SlSERAT1;1 was expressed in the mitochondria, as indicated by the green fluorescence signal of SlSERAT1;1-GFP co-localizing with the red fluorescence signal of a mitochondria marker (mt-rk). Both SlSERAT2;1 and SlSEART2;2 were expressed in chloroplasts, as indicated by their signals merging with the autofluorescence of chlorophyll. Finally, SlSERAT3;1 was localized in the cytosol, as its signal filled the whole cytoplasm (Figure 3).

As sometimes it is hard to discriminate the localization signals between the cytoplasm and the endoplasmic reticulum, the positioning signals of SlSERAT3;1 was co-analyzed with the chlorophyll autofluorescence and the peroxisome marker signals (red fluorescence, Supplementary Figure S2) for validation. The results showed that the signals of the chloroplast and the peroxisome were well surrounded by the SlSERAT3;1 signal, and this validated the cytoplasmic localization of SlSERAT3;1.

### Enzymatic activity assay of the tomato SERAT family proteins

**Figure 4:**
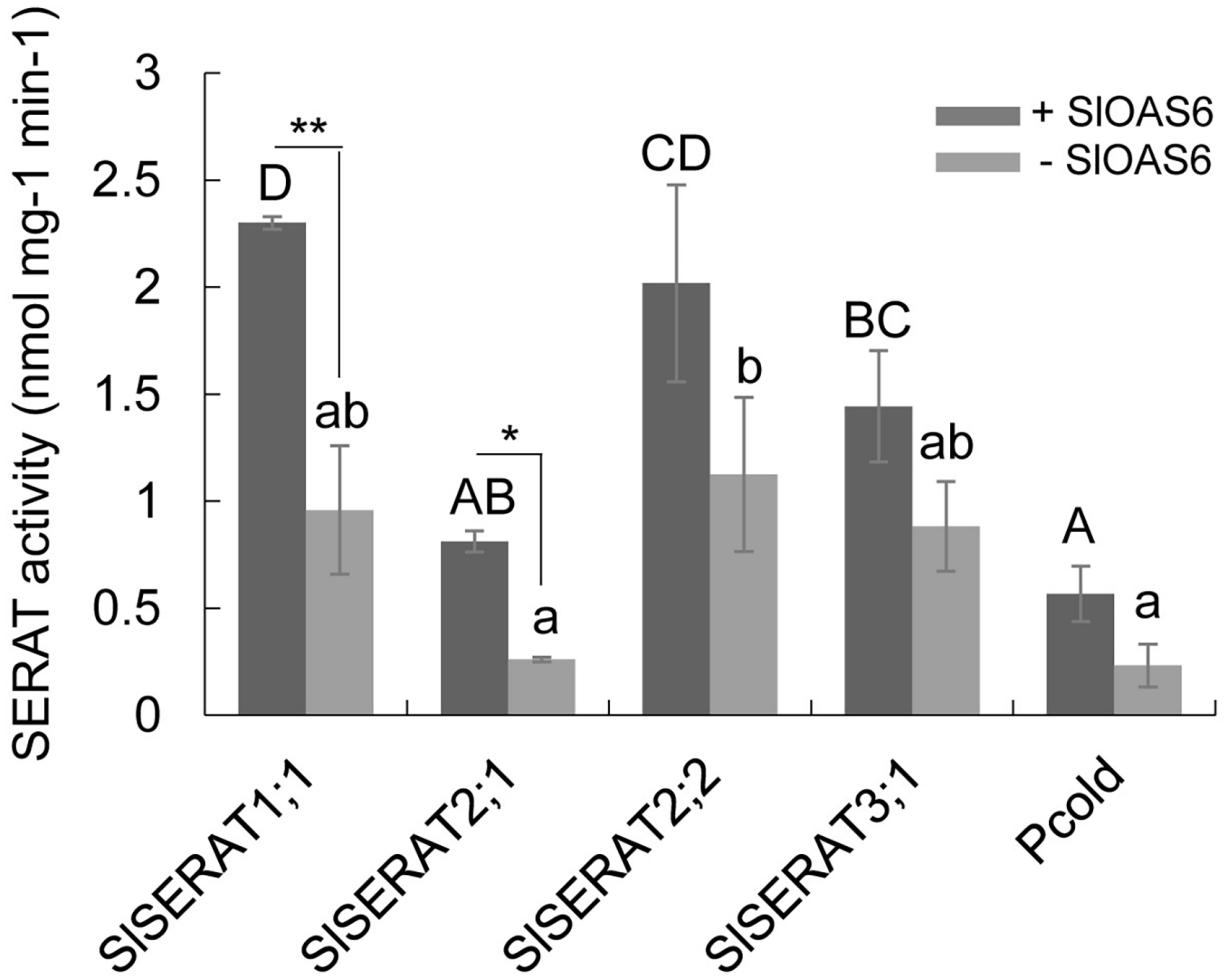
Serine acetyltransferase (SERAT) activity assays of the recombinant SlSERAT proteins in the presence (+) or the absence (−) of the OASTL protein (SlOAS6). Different letters indicate significant differences calculated by SPSS19.0 software. Letters in the upper case were used to indicate significant differences (*p* < 0.05) in the activities of different SlSERAT proteins in the presence of SlOAS6, while letters in the lower case was used to indicate significant differences (*p* < 0.05) in the activities of different SlSERAT proteins without the presence of SlOAS6. ^*^p < 0.05 and ^**^p < 0.01 indicate significant differences in the activity of the same SlSERAT protein with, or without, the presence of the OASTL protein.

To analyze the catalytic activity of these SlSERATs directly, His-tagged recombinant proteins of these tomato SERATs were first expressed in *E. coli*, purified with a Ni-NTA agarose column, and detected on SDS-PAGE gels to check their purity. As shown in Supplementary Figure S3, only one band was clearly observed for each protein. Then, the purified recombinant proteins were used to conduct the enzymatic activity assays. All the tomato SERAT proteins, except SlSERAT2;1, presented weak to moderate abilities to catalyze the biosynthesis of OAS in the absence of a OASTL protein. Among them, SlSERAT2;2 possessed the highest catalyzing ability. When a tomato OASTL protein (SlOAS6) was added to the reactions, the catalyzing abilities of all the SlSERATs increased, with the activities of SlSERAT1;1 and SlSERAT2;1 increasing significantly. In the presence of SlOAS6, SlSERAT1;1 showed the strongest ability to catalyze the formation of OAS, followed by SlSERAT2;2 and SlSERAT3;1. Although the activity of SlSERAT2;1 was greatly increased with the addition of OASTL, its catalytic ability was still weak compared with those of the other SERAT family members in tomato (Figure 4).

### Spatiotemporal expression patterns of the *SlSERAT* genes during development

**Figure 5:**
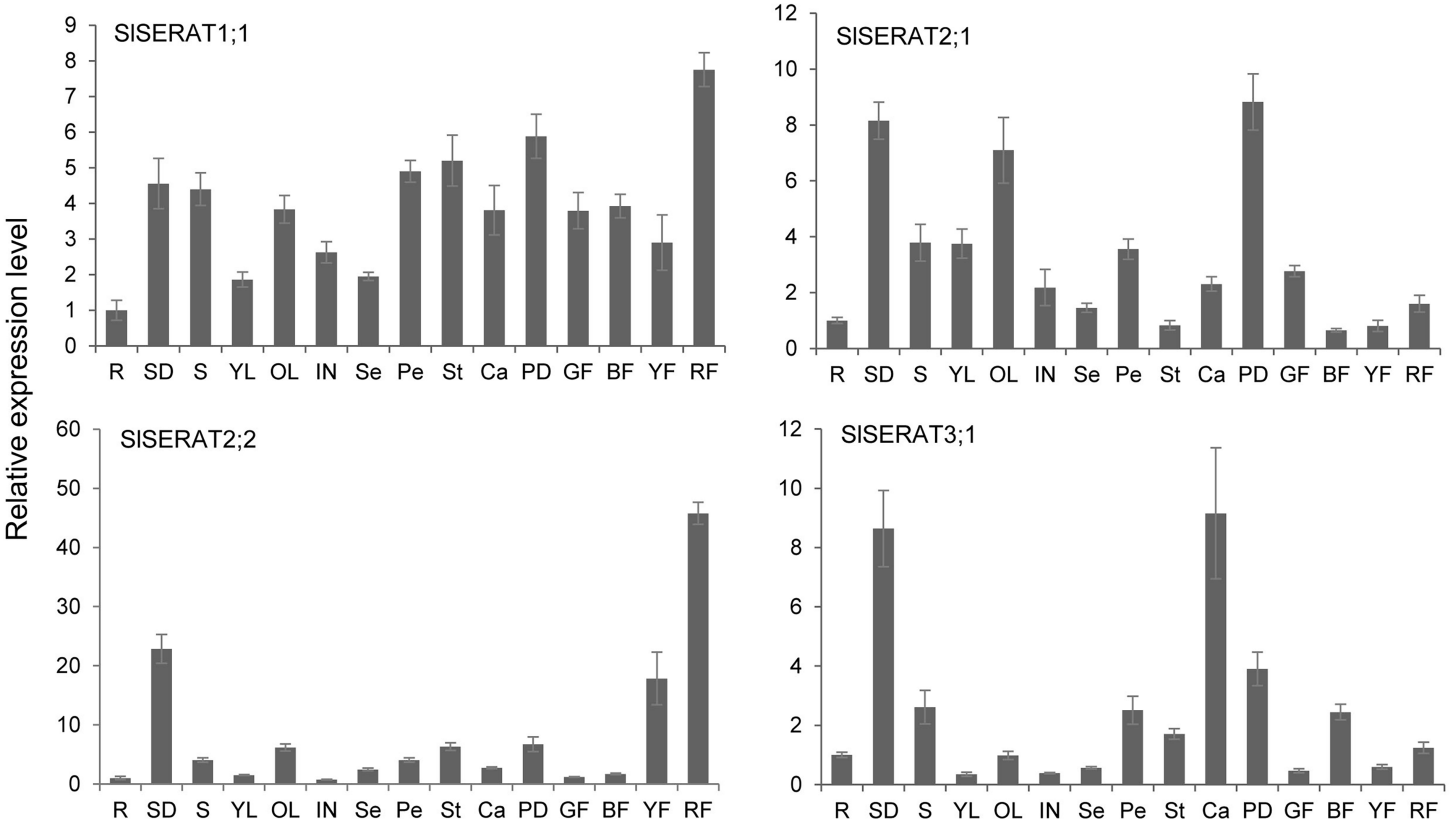
Expression pattern analyses of the SlSERAT genes during plant development. RF, red fruit; YF, yellow fruit; BF, breaker fruit; GF, immature green fruit; PD, pedicel; Ca, carpel; St, stamen; Pe, petal; Se, sepal; In, inflorescence; OL, old leaves; YL, young leaves; S, stem; SD, seedling; and R, root. The relative expression levels are fold-change values compared with root. The SAND gene was used as an endogenous control.

To further access the potential functions of these *SERAT* family genes in tomato, their tissue-specific expression patterns were investigated during plant development (Figure 5). *SlSERAT1;1* was the most widely expressed member, and its transcripts were more evenly and robustly distributed in all the tested tissues in comparison with those of the other genes. Although the proteins encoded by *SlSERAT2;1* and *SlSERAT2;2* belonged to the same subclade phylogenetically, their expression patterns were different. *SlSERAT2;2* was specifically and highly expressed in later stages of fruit development (yellow and red fruits) and in seedlings, but was relatively lowly expressed in other vegetative and reproductive tissues. *SlSERAT2;1* was relatively highly expressed in the vegetative tissues and the pedicel, but its transcription was also detected in the root, other reproductive organs, and fruits at different developmental stages. The *SlSERAT3;1* gene also had a unique expression pattern, with its highest transcription levels occurring in the seedlings and carpels.

Since the cis-elements around the promoter regions might involve in the expression pattern regulation of genes. The 3,000 bp upstream regions of all the *SlSERAT* genes were also analyzed. The results showed that light responsiveness related cis-elements were the most abundant motifs among all the upstream regions of the four *SlSERAT* genes. Phytohormone responsiveness related cis-elements could also be found in their upstream regions, among which *SlSERAT1;1* possessed the responsiveness cis-elements of five hormones (ABA, Auxin, JA, GA, and SA), while *SlSERAT3;1* only possessed the responsiveness cis-element of one hormone (ABA). For *SlSERAT2;1* and *SlSERAT2;2*, their upstream regions both contained the responsiveness cis-elements of auxin and GA. However, for *SlSERAT2;1*, it might also be regulated by ABA, while for *SlSERAT2;2*, JA and SA might be its specific regulators. Moreover, it was interesting to see that in the upstream of *SlSERAT2;2*, endosperm expression (GCN_4 motif) and zein metabolism regulation (O_2_ site) related cis-regulatory elements were found (Supplementary Figure S4). As *SlSERAT2;2* was highly expressed in the fruits, especially in late stages of fruit development (Figure 5), its expression was analyzed in seeds and flesh at different developmental stages. The result showed that, in early stage of fruit development (within 2 weeks after fertilization), there were more *SlSERAT2;2* transcripts in the seeds. However, in later developmental stages, *SlSERAT2;2* presented higher expression in the flesh, suggesting that *SlSERAT2;2* might have different roles in different stages of fruit development (Supplementary Figure S5).

### Responses of *SlSERAT* transcription to abiotic stresses

**Figure 6:**
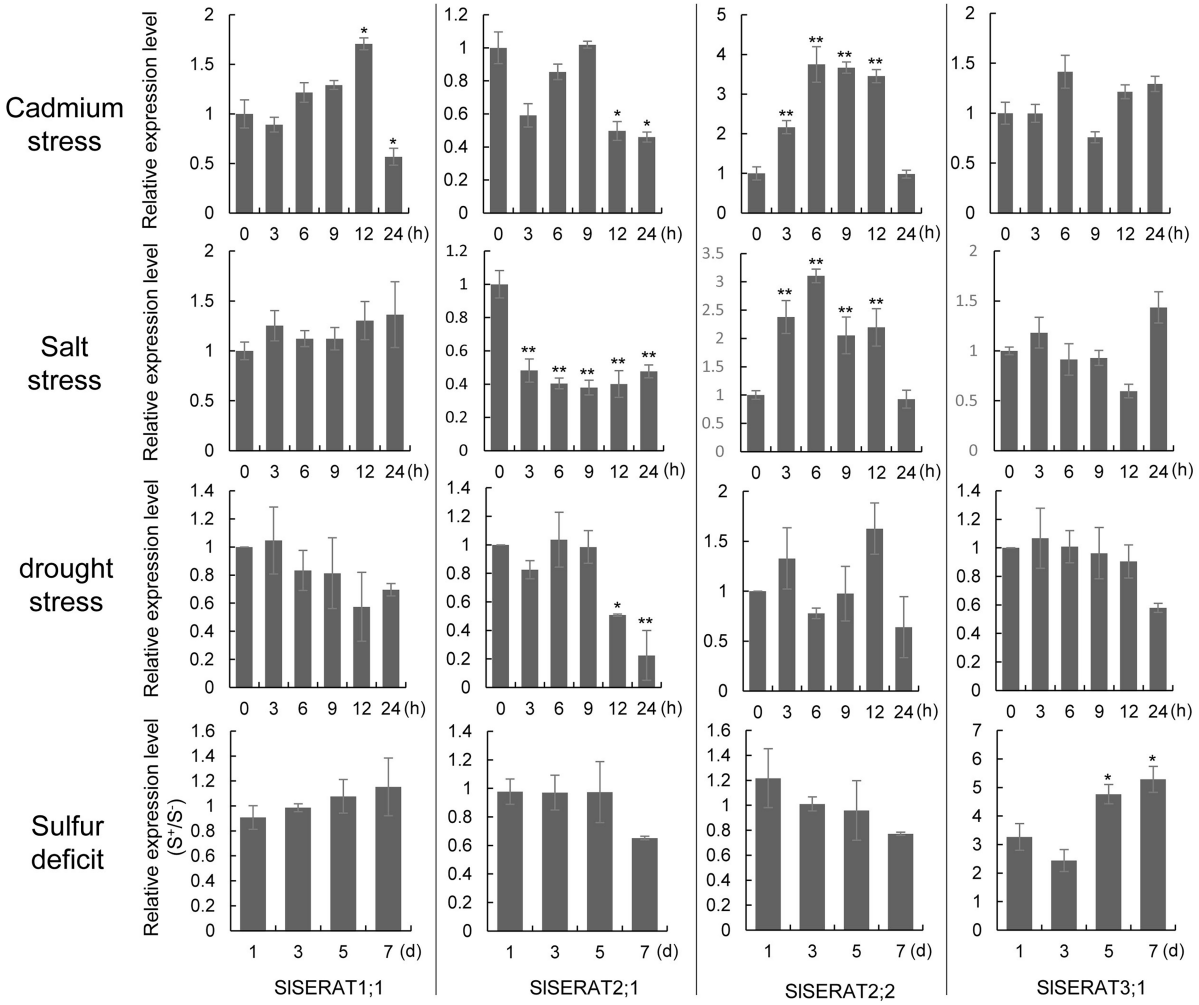
Expression level changes of the SlSERAT genes under different stresses. The tomato ACTIN gene was used as the internal control. For cadmium, salt and drought stresses, the relative expression levels were fold-change values compared with the untreated materials (0 h). For the sulfur-deficit assay, the relative expression levels were fold-change values compared with the samples grown on +S medium at every detection time point. ^*^p < 0.05 and ^**^p < 0.01 indicate statistically significant differences.

Because, in plants, SERAT proteins are the rate-limiting enzymes during Cys biogenesis, and Cys and its downstream metabolites play important roles during environmental adaption, the transcriptional changes of these tomato *SERATs* under different abiotic stresses were investigated (Figure 6). Genes in subgroup 2 (*SlSERAT2;1* and *SlSERAT2;2*) were the most active *SERATs* in response to abiotic stresses. Under both cadmium and salt stresses, the expression of *SlSERAT2;2* was upregulated from 3 h after treatment, maintained at a high expression level until 12 h after treatment, and then decreased. However, the transcription of *SlSERAT2;1* was downregulated by these two stresses. Reductions in *SlSERAT2;1* transcript were detected after 12 h under cadmium stress, while under salt stress, its transcription began to decrease at 3 h after treatment and was then maintained at a low expression level. Under drought stress, *SlSERAT2;1* was the only regulated gene, and its expression began to decrease at 12 h after treatment, though the drought-inducibility related cis-elements (MBS) were placed in the upstream regions in all the *SlSERAT* genes except *SlSERAT2;2* (Supplementary Figure S4). Finally, *SlSERAT3;1* was the only gene that responded to sulfur-deficit conditions, with its transcription being upregulated from 1 day after treatment, and its expression was significantly upregulated after 5 days.

As the SlSERAT genes from subgroup 2 were active players under stresses, and proteins encoded by these two genes were both localized in the chloroplast, we further compared the SERAT activity changes in the whole cell and the chloroplast under salt stress within 24 h after treatment. We found that the total SERAT activity decreased after salt treatment, especially at 12 h. Then at 24 h after treatment, the total SERAT activity increased slightly, but still lower than that at 0 h. Meanwhile, in the chloroplast, the SERAT activity also decreased in the first 12 h after treatment. However, at 24 h, the SERAT activity was greatly increased, which might come from the increased transcription of *SlSERAT2;2* before 24 h (Supplementary Figure S6).

### Functional analysis of *SlSERAT1;1* in tomato

As mentioned above, SlSERAT1;1 exhibited the highest activity with the presence of an OASTL protein, and localized in the mitochondrion, an organelle which has been shown to play essential roles in the biogenesis of OAS in other plant (Watanabe et al., 2008). To explore the effect of *SlSERAT1;1* on OAS and cysteine biosynthesis *in vivo*, the loss-of-function and the over-expression transgenic lines were generated for *SlSERAT1;1*.

**Figure 7:**
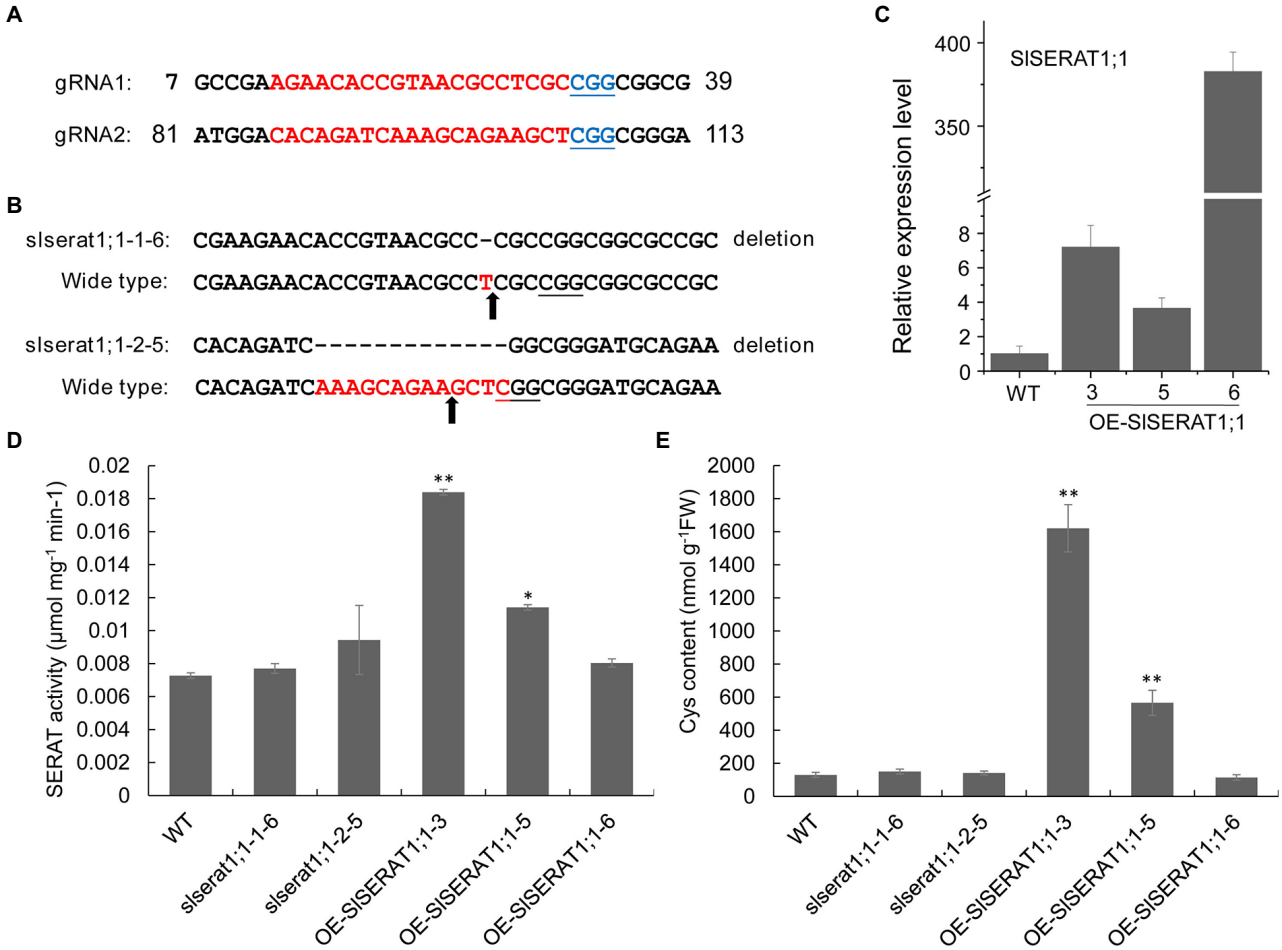
Function analysis of SlSERAT1;1 in the loss-of-function and over-expression lines. **(A)** gRNA design for the gene editing of SlSERAT1;1. the gRNA target and the protospacer-adjacent motif (PAM) are indicated in red and blue, respectively. **(B)** The mutation patterns in slserat1;1-1-6 and slserat1;1-2-5. The PAM position was underlined, and the cleavage sites were indicated by arrowheads. **(C)** The expression levels of SlSERAT1;1 in the over-expression (OE-SlSERAT1;1) lines. **(D)** SERAT activity assays in loss-of-function and over-expression lines of SlSERAT1;1. **(E)** Cys content analysis in the transgenic plants.

For the generation of loss-of-function lines, two gRNAs, targeting different positions of *SlSERAT1;1*, were designed (Figure 7A), then they were inserted into the CRISPR/Cas 9 vector (pKI1.1R) separately, and introduced into the tomato genome through Agrobacterium mediated transformation. Two homozygous mutants (*slserat1;1-1-6* and *slserat1;1-2-5*) were obtained in the T1 Plants. For *slserat1;1-1-6*, a single base T deletion occurred near the cleavage site, while in *slserat1;1-2-5*, a deletion of 13 bases (AAAGCAGAAGCTC) happened just upstream the PAM sequence (Figure 7B). In *slserat1;1-1-6*, the single-base deletion led to a severe frameshift mutation, and the coding domain of *SlSERAT1;1* was completely abolished, while in *slserat1;1-2-5*, the first 35 amino acids in the N-terminus were missing or changed compared to that in the wide type (Supplementary Figure S7). The expression level changes of *SlSERAT1;1* in these *slserat1;1* mutants were analyzed. It was interesting to find that the transcription of *SlSERAT1;1* was downregulated in these mutants (Supplementary Figure S8). Meanwhile, the over-expression transformats of *SlSERAT1;1* were also generated using the 35S promoter. Three transgenic lines were found to show increased *SlSERAT1;1* transcript, and especially, in OE-*SlSERAT1;1*-6, the expression was increased more than 300 times (Figure 7C).

Phenotypically, no obvious changes could be observed in the loss-of-function and over-expression lines, so the SERAT activity and the Cys content were further analyzed to access the function of *SlSERAT1;1 in vivo* (Figures 7D,E). In the loss-of-function lines, both the SERAT activity and the Cys content did not show significant changes. However, in the over-expression lines, the abilities to produce OAS and Cys were both improved. It was interesting to see that it was OE-*SlSERAT1;1*-3, but not OE-*SlSERAT1;1*-6 having the highest SERAT activity and the Cys content. Actually, in OE-*SlSERAT1;1*-6, the two indexes were not significantly changed, indicating that co-suppression in the protein level might happen in the OE-*SlSERAT1;1*-6 line.

## Discussion

**Figure 8:**
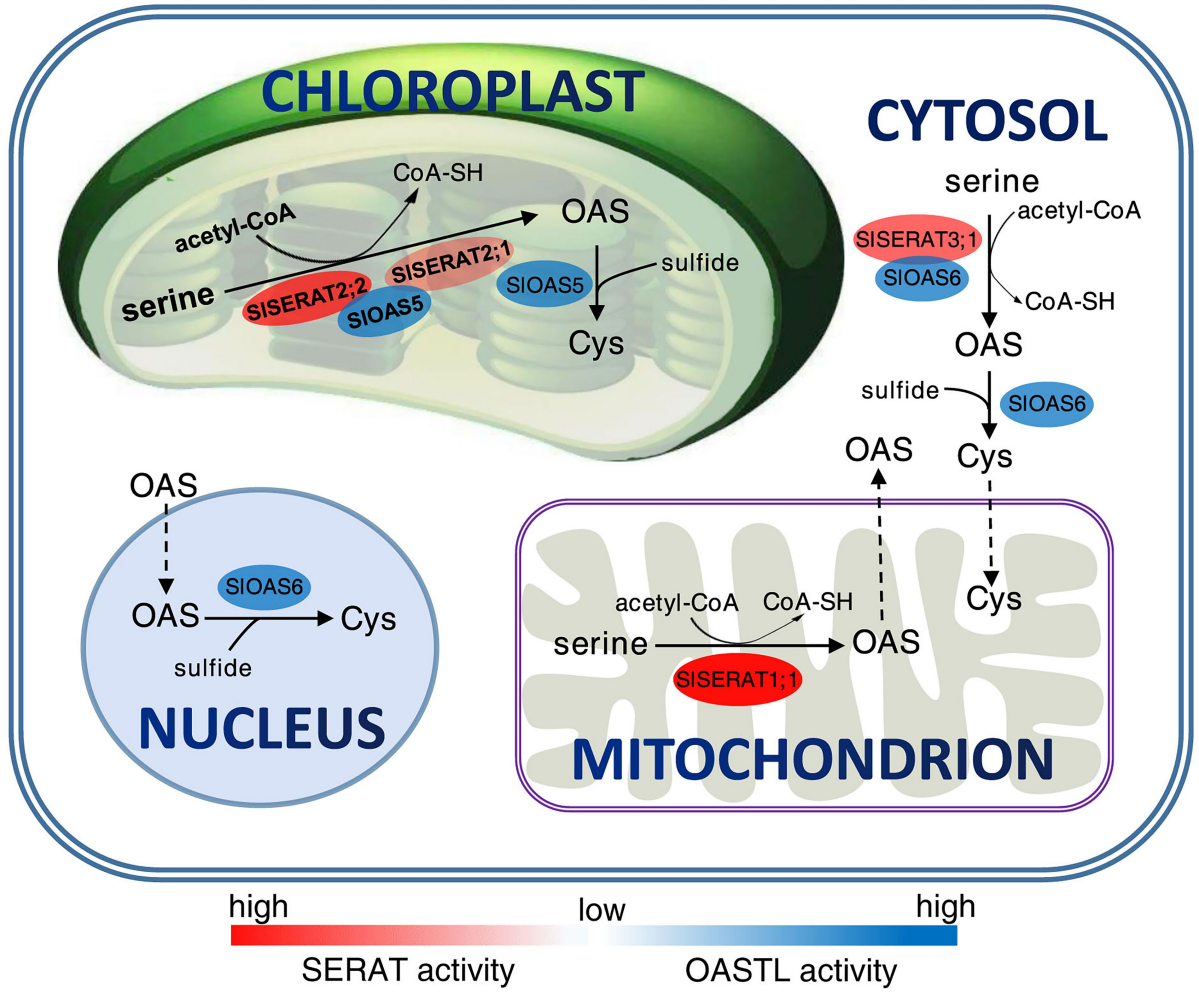
Main pathways surrounding the production and transport of the cysteine (Cys) and O-acetylserine (OAS) within a tomato cell. The SERAT proteins are red-colored, and the OASTL proteins are blue-colored. The color strength behind each enzyme indicates their relative activity detected *in vitro*. The dotted arrows show the predicted transport routes of Cys and OAS in a tomato cell based on the evidences obtained from the slsearat1;1 mutants and the nucleus localization of SlOAS6.

Cys synthesis is an essential regulatory step during the process of sulfur assimilation. In the biosynthesis of Cys, SERAT catalyzes the rate-limiting step and thus is a critical factor for sulfur metabolism and usage in plants. To date, enzymes in the SERAT family have only be systematically analyzed in fewer plant species (Watanabe et al., 2008; Tavares et al., 2015). Comprehensive investigations of SERAT proteins in other species, especially economically important crops, are still lacking. Here, four *SERAT* family genes were identified from the tomato genomic database, and they were all cloned and characterized (Figure 8). This work will help to decipher the sulfur-related metabolism in tomato, and hence make a foundation for further relative biotechnical manipulating in this economic crop.

### The conservation and specificity of the tomato SERAT proteins

Phylogenetically, proteins encoded by these four tomato *SERAT* genes were classified into three groups. In each group, subgroup members shared similar gene structures, and this feature was the same as reported in other species.

Despite this, the subcellular localization of the tomato SERAT proteins was not conserved with their homologs in other species, especially in group 1 and group 2. In tomato, SlSERAT1;1 was localized in mitochondria, while SERAT proteins from the same subgroup in *Arabidopsis* and grapevine are localized in the cytosol (Kawashima et al., 2005; Tavares et al., 2015). For proteins in subgroup 2, SlSERAT2;1 and SlSERAT2;2 were both localized in the chloroplast, however in Arabidopsis and grapevine, members in this subgroup can also be expressed in mitochondria, except the chloroplast (Watanabe et al., 2008; Tavares et al., 2015).

Regarding the functions, the catalytic activity of the SERAT proteins seemed to have more relevance with their subcellular localizations. No matter in *Arabidopsis*, grapevine, or tomato, SERAT members positioned in the mitochondria possessed the highest activity (Watanabe et al., 2008; Tavares et al., 2015). And this seems to be in consistent with a report that the Serine (Ser) production in the mitochondria may be the major source for Ser supply in the photosynthetic tissues (Voll et al., 2006). Despite this, the performance of SlSERAT3;1 was totally different from its homologs in other plant species. The activity of SlSERAT3;1 was relatively strong, being even greater than that of SlSERAT2;1 (Figure 4), which was different from its homologs in other plant species that have relatively lower abilities to catalyze the formation of OAS (Kawashima et al., 2005; Watanabe et al., 2008; Tavares et al., 2015). Additionally, the activity of SlSERAT3;1 could also be increased by the addition of OASTL protein, although not to a significant level (Figure 4). The performance of SlSERAT3;1 on the catalyzing activity might be explained by its special amino acid sequences at the C-terminus. The SlSERAT3;1 sequence was highly similar to its Arabidopsis counterpart, and most notably, it also had an extended C-terminus, which had been shown to have negative roles in the interaction between SERAT and OASTL proteins (Tavares et al., 2015). However, the essential C-terminal Ile was retained (Figure 2). Numerous studies have reported that the C-terminus of a SERAT protein is bifunctional, acting in both catalysis and interactions with OASTL proteins (Wirtz et al., 2001; Yi et al., 2013). The C-terminal Ile functions as an anchor for OASTL proteins in these interactions and, thus, may play a fundamental role in CSC formation (Campanini et al., 2005; Francois et al., 2006). In *Arabidopsis*, both SERAT3;1 and SERAT3;2 are mutated at this amino acid site, having the Ile changed to Asp and Val, respectively (Figure 2), and hence affect their activities. Here, in tomato, it was possible that though the C-terminus extension of SlSERAT3;1 weakened its interaction with the OASTL proteins, the remaining Ile residue still promoted the interaction, and thus resulted in a weak or instant formed CSC complex between SlSERAT3;1 and OASTL proteins to enhance the activity of SlSERAT3;1. Because the interactions between SlSERAT3;1 and the OASTL proteins were weak or instant, they would be hard to detect in the yeast two-hybrid system; therefore, we did not find their interactions in our previous study (Liu et al., 2019). Moreover, we also found that the Ile residue at this position was conserved in all the analyzed SERAT3 members from Solanaceae family, indicating that they might also interact with OASTL proteins, and therefore have higher activities, but this needs further confirmation. In addition, while in most cases, the interaction with OASTL increased the SERAT activity, the extent of the increase varied among different SERATs (Figure 4; Tavares et al., 2015). In *E. coli* and *Salmonella typhimurium*, the complexes formed by SERAT and OASTL proteins have no effects on SERAT activity levels, but only inhibit the activities of the OASTL proteins (Campanini et al., 2005). As most of the reported plant SERAT proteins have a catalytic ability without the assistance of OASTL proteins, the promotive effect of the CSC on the SERAT proteins has been regarded as an “in-passing” function in some documents (Kawashima et al., 2005; Tavares et al., 2015). Nevertheless, such an evolution of the regulatory function of the CSC has made the related metabolic pathways more efficient and economical in plants.

### Functional diversification of the tomato SERAT genes under different stresses

In plants, thiols play a central role in abiotic stress responses (Mulet et al., 2004; Liu et al., 2006; Na and Salt, 2011). Cys is the first sulfur-containing organic molecule generated by plants. Cys *per se* and its derivatives, such as glutathione and phytochelatins, play important roles in plant resistance to multiple stresses (Romero et al., 2014). As the rate-limiting enzyme during Cys generation, SERAT is involved in responses to a range of abiotic stresses. Moreover, to balance plant growth and stress tolerance, *SERAT* genes may function differentially in response to different stresses (Ahmad et al., 2016). In this work, we found that the chloroplast-positioned tomato SERAT members (SlSERAT2s) were the main responders to abiotic stresses in tomato. And correspondingly, we found that it was the chloroplast SERAT activity, but not the total SERAT activity increased at 24 h after salt stress treatment, indicating an delicate regulation by the plant under stresses. To make things more complicate and interesting, we also found that the SlSERAT2s presented opposite expression responses to cadmium and salt stresses. The transcription of SlSERAT2;2 was greatly upregulated by these two stresses from 3 h after exposure, while the expression of SlSERAT2;1 was downregulated, especially after 12 h, suggesting functional diversification in the same subgroup of the SlSERAT family. Moreover, considering the huge gap on the enzymatic activity between the two SlSERAT2s, and the fact that they can both interact with the OASTL proteins, it will be interesting to see whether these two SlSERAT2s act antagonistically under stress by competing the same OASTL protein. Comparably, the responses of these four genes to drought stress were moderate, with only SlSERAT2;1 showing a reduction in the transcription at 12 h after treatment. The decrease in SlSERAT2;1 expression in later stages might represent a retardation of growth under drought conditions. Thus, plants may have different coping strategies in response to osmotic stress, ion toxicity or their combination.

### Function redundancy of the *SlSERAT* genes and their latent application value in plant S-assimilation modification

In Arabidopsis, SERAT activity is vital for plant viability (Krueger et al., 2009); however, no visible phenotype could be detected in the single mutants of Arabidopsis SERAT genes, indicating redundancy exists among SERAT members within a single plant species (Watanabe et al., 2008). Here, in this study, the loss-of-function transformat of SlSERAT1;1 (*slserat1;1-1-6*) showed no phenotype defects, and the SERAT activity and Cys contents were not changed (Figure 7), suggesting that function redundancy was also applicable to the tomato SERAT members. Actually, as OAS is an important intermediate and a potential signaling molecule during the formation of Cys and the sulfur-assimilation system, it is not surprising that function redundancy of the SERAT proteins was used by the plant to guarantee its OAS supply. However, on the other hand, plant also has high tolerance to the concentration changes of OAS. In Arabidopsis, it has been reported that no remarkable phenotypic changes could be noticed in the quadruple mutants Q1;1 and Q2;2, though the OAS levels were significantly decreased in these mutants (Watanabe et al., 2008). Moreover, it is also well known that overexpression of SERAT members in plants could enhance the S-assimilation without negative impact on plant growth (Sirko et al., 2004; Xiang et al., 2018), suggesting the tolerance of plants to high OAS levels. However, the mechanism underlying the tolerance still needs further investigation. In this work, overexpression of SlSERAT1;1 also increase the SERAT activity and the Cys content without affecting the phenotype, adding the evidence that SERAT is an ideal target for S-assimilation manipulating in tomato.

In this work, it is surprising that though SlSERAT1;1 was mitochondria-localized and showed robust catalyzing activity *in vitro*, its mutant plants showed no changes in both the SERAT activity and Cys contents, which is different from its counterpart (SERAT2;2) in Arabidopsis (Krueger et al., 2009). The lack of a true OASTL in the mitochondria in tomato might be an explanation for this. In tomato, there are only two OASTL members (SlOAS5 and SlOAS6) possessing the ability to catalyze the last step of Cys biosynthesis, and neither of them is positioned in the mitochondria (Liu et al., 2019; Figure 8). Thus, mitochondria might not be an important compartment for Cys biosynthesis in tomato, and the cytosol and the plastid might have evolved strong biosynthesis systems for both OAS and Cys. In the meantime, due to the efficient transport of Cys, OAS and sulfide between cytosol and organelles (Heeg et al., 2008), the mitochondria in the *slserat1;1* mutants could also obtain enough Cys for the protein biosynthesis, and hence maintain its function and the normal plant phenotype.

Furthermore, in this study, we also found that high transcription of the overexpressed SERAT genes did not necessarily lead to the improvement of the SERAT activities, reminding that co-suppression cannot be neglected in the overexpression transformats, especially those with abnormally high expression levels of the transformed genes.

## Data availability statement

The original contributions presented in the study are included in the article/Supplementary material, further inquiries can be directed to the corresponding author.

## Author contributions

DL, YP, and ML designed the experiments and wrote the manuscript. DL, ML, TG, JL, YX, YH, LW, DZ, LZ, ZL, ZJ, and YP were performed the experiments. DL and YP analyzed the data. All authors performed the experiments, contributed to the article, and approved the submitted version.

## Funding

This work is funded by the Fundamental Research Program of Shanxi Province (20210302123450) and the National Natural Science Foundation of China (32172550 and 31501772).

## Conflict of interest

The authors declare that the research was conducted in the absence of any commercial or financial relationships that could be construed as a potential conflict of interest.

## Publisher’s note

All claims expressed in this article are solely those of the authors and do not necessarily represent those of their affiliated organizations, or those of the publisher, the editors and the reviewers. Any product that may be evaluated in this article, or claim that may be made by its manufacturer, is not guaranteed or endorsed by the publisher.

## Footnotes

^1^ http://bioinformatics.psb.ugent.be/webtools/plantcare/html/

^2^ http://crispr.dbcls.jp/

^3^ http://rna.urmc.rochester.edu/RNAstructureWeb/Servers/Predict1/Predict1.html

## References

[ref1] Ahmad N. Malagoli M. Wirtz M. Hell R. (2016). Drought stress in maize causes differential acclimation responses of glutathione and sulfur metabolism in leaves and roots. BMC Plant Biol. 16:247. doi: 10.1186/s12870-016-0940-z 27829370 PMC5103438

[ref2] Ba Y. R. Zhai J. L. Yan J. P. Li K. Z. Xu H. I. (2021). H2S improves growth of tomato seedlings involving the MAPK signaling. Sci. Hortic. 288:110366. doi: 10.1016/j.scienta.2021.110366

[ref3] Campanini B. Speroni F. Salsi E. Cook P. F. Roderick S. L. Huang B.. (2005). Interaction of serine acetyltransferase with O-acetylserine sulfhydrylase active site: evidence from fluorescence spectroscopy. Protein Sci. 14, 2115–2124. doi: 10.1110/ps.051492805 15987896 PMC2279323

[ref4] Exposito-Rodriguez M. Borges A. A. Borges-Perez A. Perez J. A. (2008). Selection of internal control genes for quantitative real-time RT-PCR studies during tomato development process. BMC Plant Biol. 8:131. doi: 10.1186/1471-2229-8-131 19102748 PMC2629474

[ref5] Fang H. Jing T. Liu Z. Zhang L. Jin Z. Pei Y. (2014). Hydrogen sulfide interacts with calcium signaling to enhance the chromium tolerance in *Setaria italica*. Cell Calcium 56, 472–481. doi: 10.1016/j.ceca.2014.10.004 25459298

[ref6] Fang H. Liu Z. Jin Z. Zhang L. Liu D. Pei Y. (2016). An emphasis of hydrogen sulfide-cysteine cycle on enhancing the tolerance to chromium stress in *Arabidopsis*. Environ. Pollut. 213, 870–877. doi: 10.1016/j.envpol.2016.03.035 27038574

[ref7] Francois J. A. Kumaran S. Jez J. M. (2006). Structural basis for interaction of O-acetylserine sulfhydrylase and serine acetyltransferase in the Arabidopsis cysteine synthase complex. Plant Cell 18, 3647–3655. doi: 10.1105/tpc.106.047316 17194764 PMC1785398

[ref8] Freeman J. L. Salt D. E. (2007). The metal tolerance profile of Thlaspi goesingense is mimicked in *Arabidopsis thaliana* heterologously expressing serine acetyl-transferase. BMC Plant Biol. 7, 63. doi: 10.1186/1471-2229-7-63 18045473 PMC2233625

[ref9] Haas F. H. Heeg C. Queiroz R. Bauer A. Wirtz M. Hell R. (2008). Mitochondrial serine acetyltransferase functions as a pacemaker of cysteine synthesis in plant cells. Plant Physiol. 148, 1055–1067. doi: 10.1104/pp.108.125237 18753283 PMC2556817

[ref10] Harms K. von Ballmoos P. Brunold C. Hofgen R. Hesse H. (2000). Expression of a bacterial serine acetyltransferase in transgenic potato plants leads to increased levels of cysteine and glutathione. Plant J. 22, 335–343. doi: 10.1046/j.1365-313x.2000.00743.x 10849350

[ref11] Heeg C. Kruse C. Jost R. Gutensohn M. Ruppert T. Wirtz M.. (2008). Analysis of the Arabidopsis O-acetylserine(thiol)lyase gene family demonstrates compartment-specific differences in the regulation of cysteine synthesis. Plant Cell 20, 168–185. doi: 10.1105/tpc.107.056747 18223034 PMC2254930

[ref12] Hell R. Jost R. Berkowitz O. Wirtz M. (2002). Molecular and biochemical analysis of the enzymes of cysteine biosynthesis in the plant *Arabidopsis thaliana*. Amino Acids 22, 245–257. doi: 10.1007/s007260200012 12083068

[ref13] Jez J. M. Dey S. (2013). The cysteine regulatory complex from plants and microbes: what was old is new again. Curr. Opin. Struct. Biol. 23, 302–310. doi: 10.1016/j.sbi.2013.02.011 23510784

[ref14] Kawashima C. G. Berkowitz O. Hell R. Noji M. Saito K. (2005). Characterization and expression analysis of a serine acetyltransferase gene family involved in a key step of the sulfur assimilation pathway in *Arabidopsis*. Plant Physiol. 137, 220–230. doi: 10.1104/pp.104.045377 15579666 PMC548853

[ref15] Krueger S. Niehl A. Lopez Martin M. C. Steinhauser D. Donath A. Hildebrandt T.. (2009). Analysis of cytosolic and plastidic serine acetyltransferase mutants and subcellular metabolite distributions suggests interplay of the cellular compartments for cysteine biosynthesis in *Arabidopsis*. Plant Cell Environ. 32, 349–367. doi: 10.1111/j.1365-3040.2009.01928.x 19143986

[ref16] Kumar S. Kumar N. Alam N. Gourinath S. (2014). Crystal structure of serine acetyl transferase from *Brucella abortus* and its complex with coenzyme A. Biochim. Biophys. Acta 1844, 1741–1748. doi: 10.1016/j.bbapap.2014.07.009 25058332

[ref17] Kumar S. Stecher G. Tamura K. (2016). MEGA7: molecular evolutionary genetics analysis version 7.0 for bigger datasets. Mol. Biol. Evol. 33, 1870–1874. doi: 10.1093/molbev/msw054 27004904 PMC8210823

[ref18] Lescot M. Dehais P. Thijs G. Marchal K. Moreau Y. Van de Peer Y.. (2002). PlantCARE, a database of plant cis-acting regulatory elements and a portal to tools for in silico analysis of promoter sequences. Nucleic Acids Res. 30, 325–327. doi: 10.1093/nar/30.1.325 11752327 PMC99092

[ref19] Leustek T. Martin M. N. Bick J. A. Davies J. P. (2000). Pathways and regulation of sulfur metabolism revealed through molecular and genetic studies. Annu. Rev. Plant Physiol. Plant Mol. Biol. 51, 141–165. doi: 10.1146/annurev.arplant.51.1.141 15012189

[ref20] Liu M. Ju Y. L. Min Z. Fang Y. L. Meng J. F. (2020). Transcriptome analysis of grape leaves reveals insights into response to heat acclimation. Sci. Hortic. 272:109554. doi: 10.1016/j.scienta.2020.109554

[ref21] Liu D. Lu J. Li H. Wang J. Pei Y. (2019). Characterization of the O-acetylserine(thiol)lyase gene family in *Solanum lycopersicum* L. Plant Mol. Biol. 99, 123–134. doi: 10.1007/s11103-018-0807-9 30535734

[ref22] Liu D. Wang D. Qin Z. Zhang D. Yin L. Wu L.. (2014). The SEPALLATA MADS-box protein SLMBP21 forms protein complexes with JOINTLESS and MACROCALYX as a transcription activator for development of the tomato flower abscission zone. Plant J. 77, 284–296. doi: 10.1111/tpj.12387 24274099

[ref23] Liu F. Yoo B. C. Lee J. Y. Pan W. Harmon A. C. (2006). Calcium-regulated phosphorylation of soybean serine acetyltransferase in response to oxidative stress. J. Biol. Chem. 281, 27405–27415. doi: 10.1074/jbc.M604548200 16854983

[ref24] Livak K. J. Schmittgen T. D. (2001). Analysis of relative gene expression data using real-time quantitative PCR and the 2(T)(-Delta Delta C) method. Methods 25, 402–408. doi: 10.1006/meth.2001.1262 11846609

[ref25] Mulet J. M. Alemany B. Ros R. Calvete J. J. Serrano R. (2004). Expression of a plant serine O-acetyltransferase in *Saccharomyces cerevisiae* confers osmotic tolerance and creates an alternative pathway for cysteine biosynthesis. Yeast 21, 303–312. doi: 10.1002/yea.1076 15042590

[ref26] Na G. Salt D. E. (2011). Differential regulation of serine acetyltransferase is involved in nickel hyperaccumulation in *Thlaspi goesingense*. J. Biol. Chem. 286, 40423–40432. doi: 10.1074/jbc.M111.247411 21930704 PMC3220491

[ref27] Nelson B. K. Cai X. Nebenfuhr A. (2007). A multicolored set of in vivo organelle markers for co-localization studies in *Arabidopsis* and other plants. Plant J. 51, 1126–1136. doi: 10.1111/j.1365-313X.2007.03212.x 17666025

[ref28] Noji M. Inoue K. Kimura N. Gouda A. Saito K. (1998). Isoform-dependent differences in feedback regulation and subcellular localization of serine acetyltransferase involved in cysteine biosynthesis from *Arabidopsis thaliana*. J. Biol. Chem. 273, 32739–32745. doi: 10.1074/jbc.273.49.32739 9830017

[ref29] Romero L. C. Aroca M. A. Laureano-Marin A. M. Moreno I. Garcia I. Gotor C. (2014). Cysteine and cysteine-related signaling pathways in *Arabidopsis thaliana*. Mol. Plant 7, 264–276. doi: 10.1093/mp/sst168 24285094

[ref30] Saito K. (2000). Regulation of sulfate transport and synthesis of sulfur-containing amino acids. Curr. Opin. Plant Biol. 3, 188–195. doi: 10.1016/S1369-5266(00)00063-7 10837270

[ref31] Singh V. K. Mangalam A. K. Dwivedi S. Naik S. (1998). Primer premier: program for design of degenerate primers from a protein sequence. BioTechniques 24, 318–319. doi: 10.2144/98242pf02 9494736

[ref32] Sirko A. Blaszczyk A. Liszewska F. (2004). Overproduction of SAT and/or OASTL in transgenic plants: a survey of effects. J. Exp. Bot. 55, 1881–1888. doi: 10.1093/jxb/erh151 15208350

[ref33] Tabe L. Wirtz M. Molvig L. Droux M. Hell R. (2010). Overexpression of serine acetlytransferase produced large increases in O-acetylserine and free cysteine in developing seeds of a grain legume. J. Exp. Bot. 61, 721–733. doi: 10.1093/jxb/erp338 19939888 PMC2814105

[ref34] Tavares S. Wirtz M. Beier M. P. Bogs J. Hell R. Amancio S. (2015). Characterization of the serine acetyltransferase gene family of *Vitis vinifera* uncovers differences in regulation of OAS synthesis in woody plants. Front. Plant Sci. 6, 74. doi: 10.3389/fpls.2015.00074 25741355 PMC4330696

[ref35] Thompson J. D. Gibson T. J. Higgins D. G. (2002). Multiple sequence alignment using ClustalW and ClustalX. *Curr Protoc bioinformatics*, *chapter 2*, pp. unit 2 3. 10.1002/0471250953.bi0203s00 18792934

[ref36] Tsutsui H. Higashiyama T. (2017). pKAMA-ITACHI vectors for highly efficient CRISPR/Cas9-mediated gene knockout in *Arabidopsis thaliana*. Plant Cell Physiol. 58, 46–56. doi: 10.1093/pcp/pcw191 27856772 PMC5444565

[ref37] Voll L. M. Jamai A. Renne P. Voll H. McClung C. R. Weber A. P. M. (2006). The photorespiratory Arabidopsis *shm1* mutant is deficient in SHM1. Plant Physiol. 140, 59–66. doi: 10.1104/pp.105.071399 16339799 PMC1326031

[ref38] Wang Y. Q. Zhang Y. X. Gao Z. P. Yang W. C. (2018). Breeding for resistance to tomato bacterial diseases in China: challenges and prospects. Hortic. Plant J. 4, 193–207. doi: 10.1016/j.hpj.2018.08.004

[ref39] Watanabe M. Mochida K. Kato T. Tabata S. Yoshimoto N. Noji M.. (2008). Comparative genomics and reverse genetics analysis reveal indispensable functions of the serine acetyltransferase gene family in *Arabidopsis*. Plant Cell 20, 2484–2496. doi: 10.1105/tpc.108.060335 18776059 PMC2570737

[ref40] Wirtz M. Berkowitz O. Droux M. Hell R. (2001). The cysteine synthase complex from plants. Mitochondrial serine acetyltransferase from *Arabidopsis thaliana* carries a bifunctional domain for catalysis and protein-protein interaction. Eur. J. Biochem. 268, 686–693. doi: 10.1046/j.1432-1327.2001.01920.x 11168407

[ref41] Wirtz M. Hell R. (2007). Dominant-negative modification reveals the regulatory function of the multimeric cysteine synthase protein complex in transgenic tobacco. Plant Cell 19, 625–639. doi: 10.1105/tpc.106.043125 17293569 PMC1867341

[ref42] Xiang X. Wu Y. Planta J. Messing J. Leustek T. (2018). Overexpression of serine acetyltransferase in maize leaves increases seed-specific methionine-rich zeins. Plant Biotechnol. J. 16, 1057–1067. doi: 10.1111/pbi.12851 29044890 PMC5902772

[ref43] Yi H. Dey S. Kumaran S. Lee S. G. Krishnan H. B. Jez J. M. (2013). Structure of soybean serine acetyltransferase and formation of the cysteine regulatory complex as a molecular chaperone. J. Biol. Chem. 288, 36463–36472. doi: 10.1074/jbc.M113.527143 24225955 PMC3868759

