# Supplementary material for: Functional characterization of the Serine acetyltransferase family genes uncovers the diversification and conservation of cysteine biosynthesis in tomato
Source: Front Plant Sci. 2022 Sep 21;13:913856. doi: 10.3389/fpls.2022.913856 (PMC9533716; doi:10.3389/fpls.2022.913856)
Supplement: Supplementary file 1 [file Data_Sheet_1.PDF]

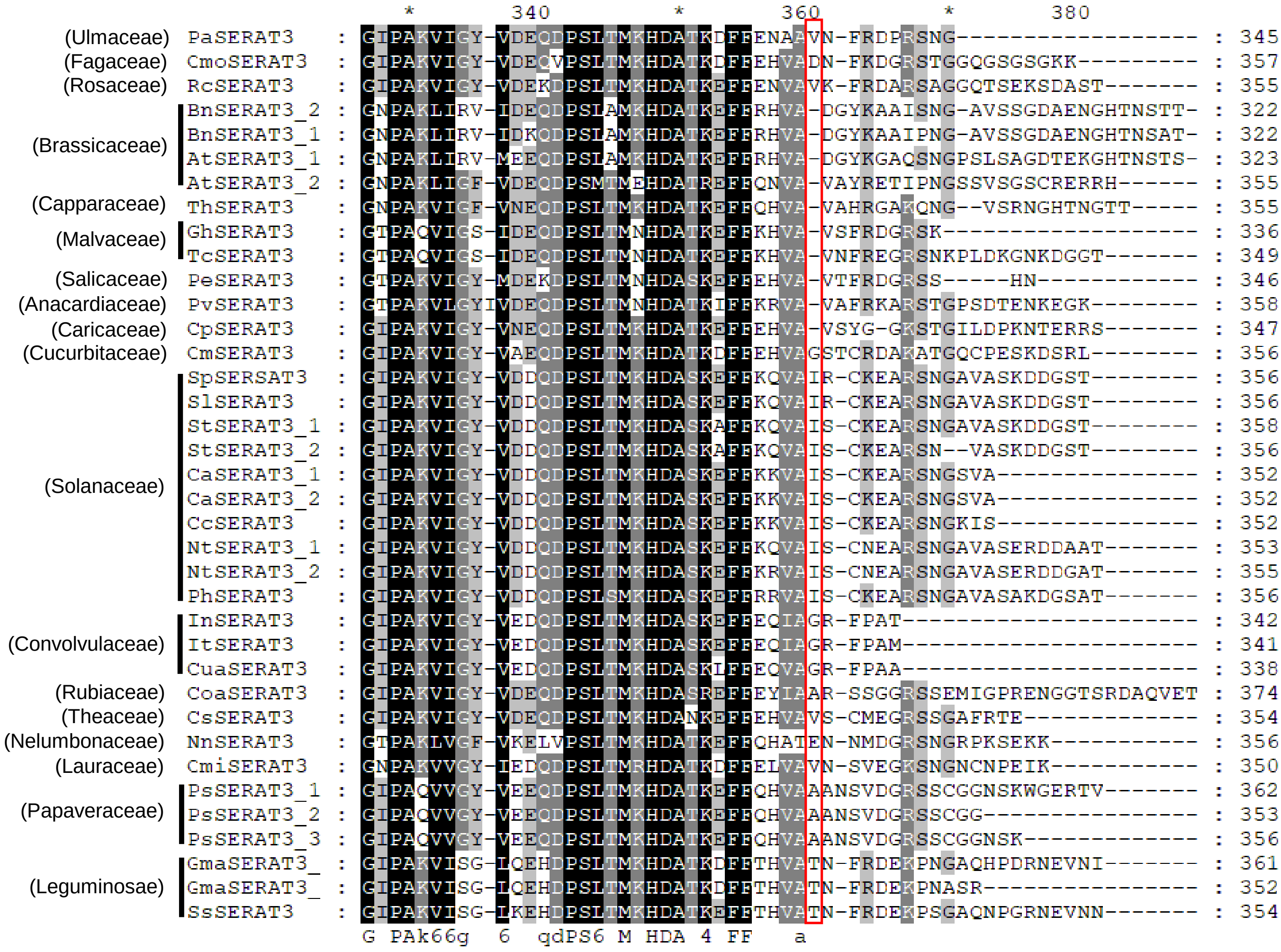

Figure S1. Alignment analysis of the C-terminus sequences of the SERAT3 subgroup members from 29 species in 18 families. Pa, *Parasponia andersonii*; Cmo, *Castanea mollissima*; Rc, *Rosa chinensis*; Bn, *Brassica napus*; At, *Arabidopsis thaliana*; Th, *Tarenaya hassleriana*; Gh, *Gossypium hirsutum*; Tc, *Theobroma cacao*; Pe, *Populus euphratica*; Pv, *Pistacia vera*; Cp, *Carica papaya*; Cm, *Cucumis melo*; Sp, *Solanum pennellii*; Sl, *Solanum lycopersicum*; St, *Solanum tuberosum*; Ca, *Capsicum annuum*; Cc, *Cajanus cajan*; Nt, *Nicotiana tabacum*; Ph, *Petunia hybrida*; In, *Ipomoea nil*; It, *Ipomoea triloba*; Cua, *Cuscuta australis*, Coa, *Coffea arabica*; Cs, *Camelina sativa*; Nn, *Nelumbo nucifera*; Cmi, *Cinnamomum micranthum*; Ps, *Papaver somniferum*; Gma, *Glycine max*; Ss, *Spatholobus suberectus*

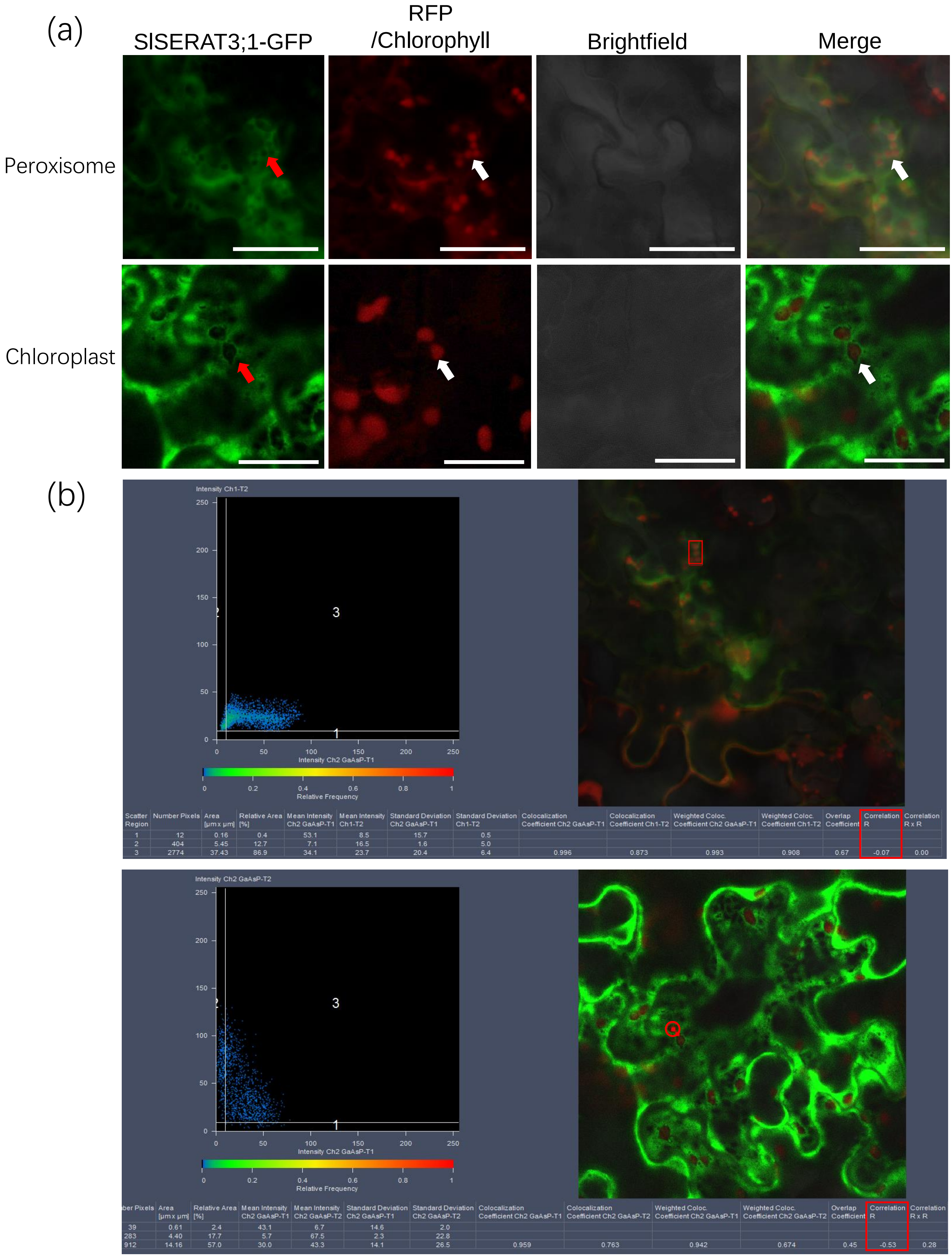

Figure S2. Co-analysis of the SlSERAT3;1 location signals with the peroxisome position marker and the autofluorescence of chlorophyll.

(a) The GPF signals of SlSERAT3;1 surrounded the fluorescence signals of the peroxisome position marker and the chlorophyll. Note the vacuoles in the SlSERAT3;1 signal pointed by red arrows in the first row.

(b) The correlation analyses between the green and the red fluorescence signals. The R values (in the red rectangle) were -0.07 and -0.53 respectively, both indicated non-colocalization.

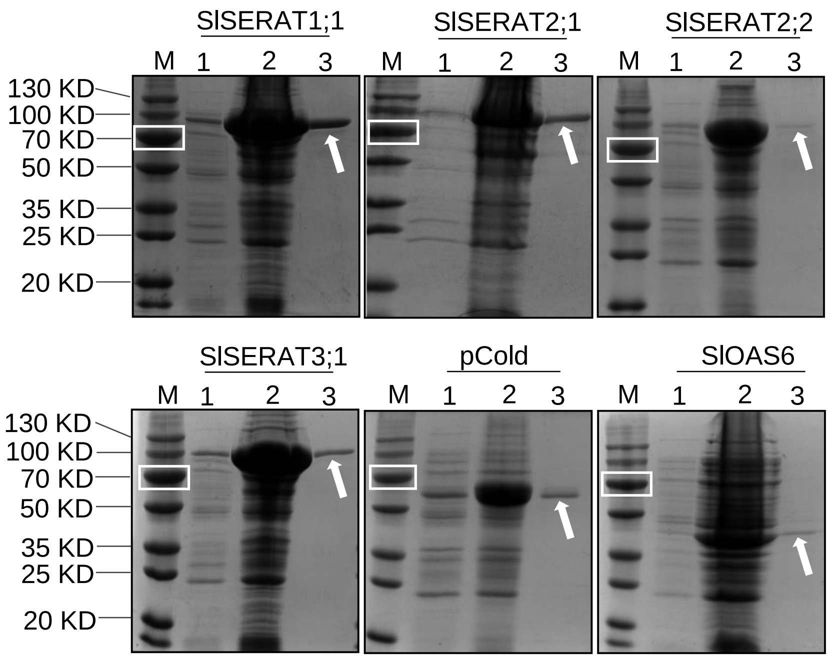

Fig. S3 SDS-PAGE analyses of the induced and purified SlSERAT proteins, the empty pCold vector, and SlOAS6. M, protein MW marker; lane 1, protein extracts from the uninduced bacteria; lane 2, protein extracts after induction by adding IPTG; lane 3, the purified proteins.

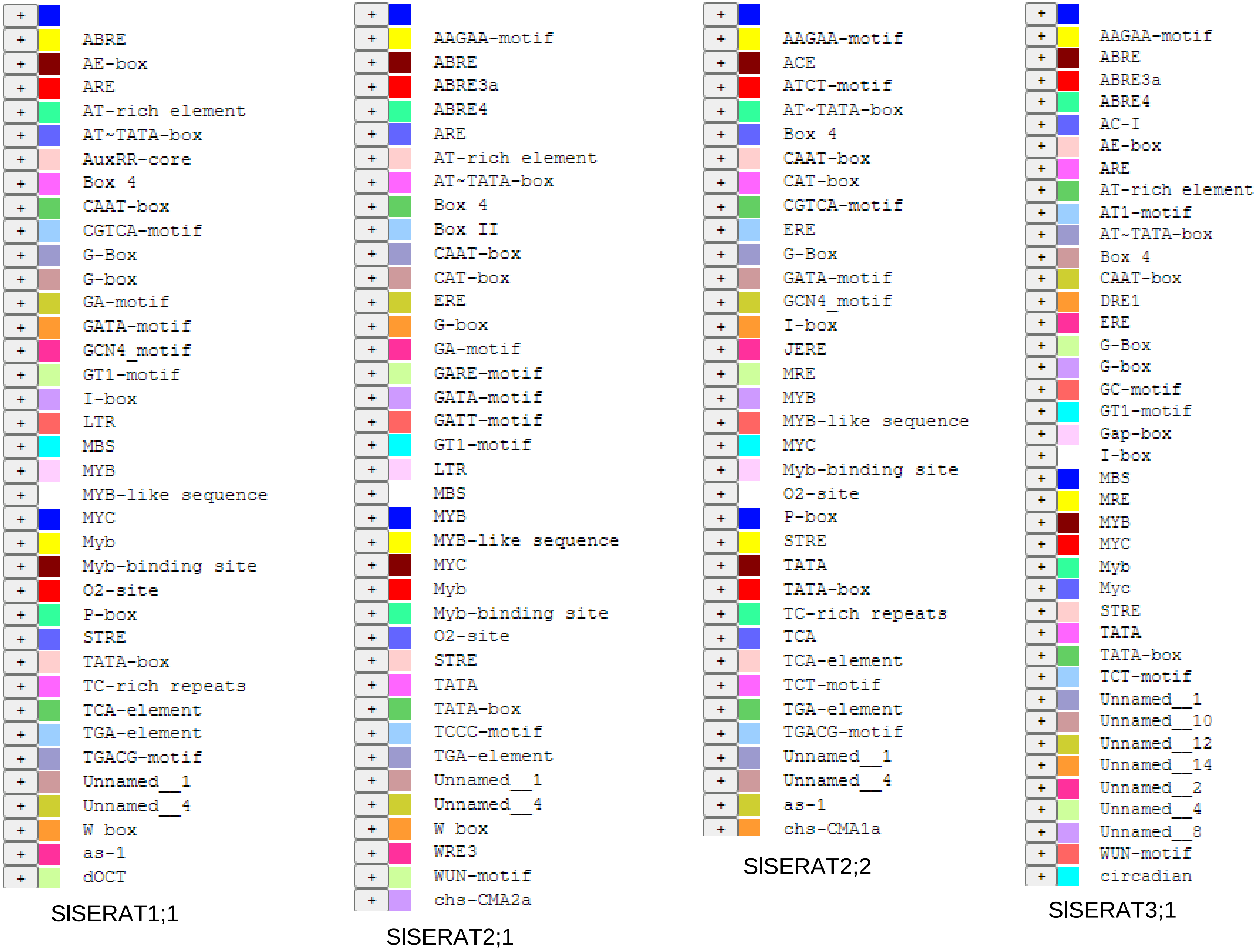

Figure S4 Cis-regulatory acting element analyses in the 3000 bp upstream regions of the tomato *SlSERAT* genes.

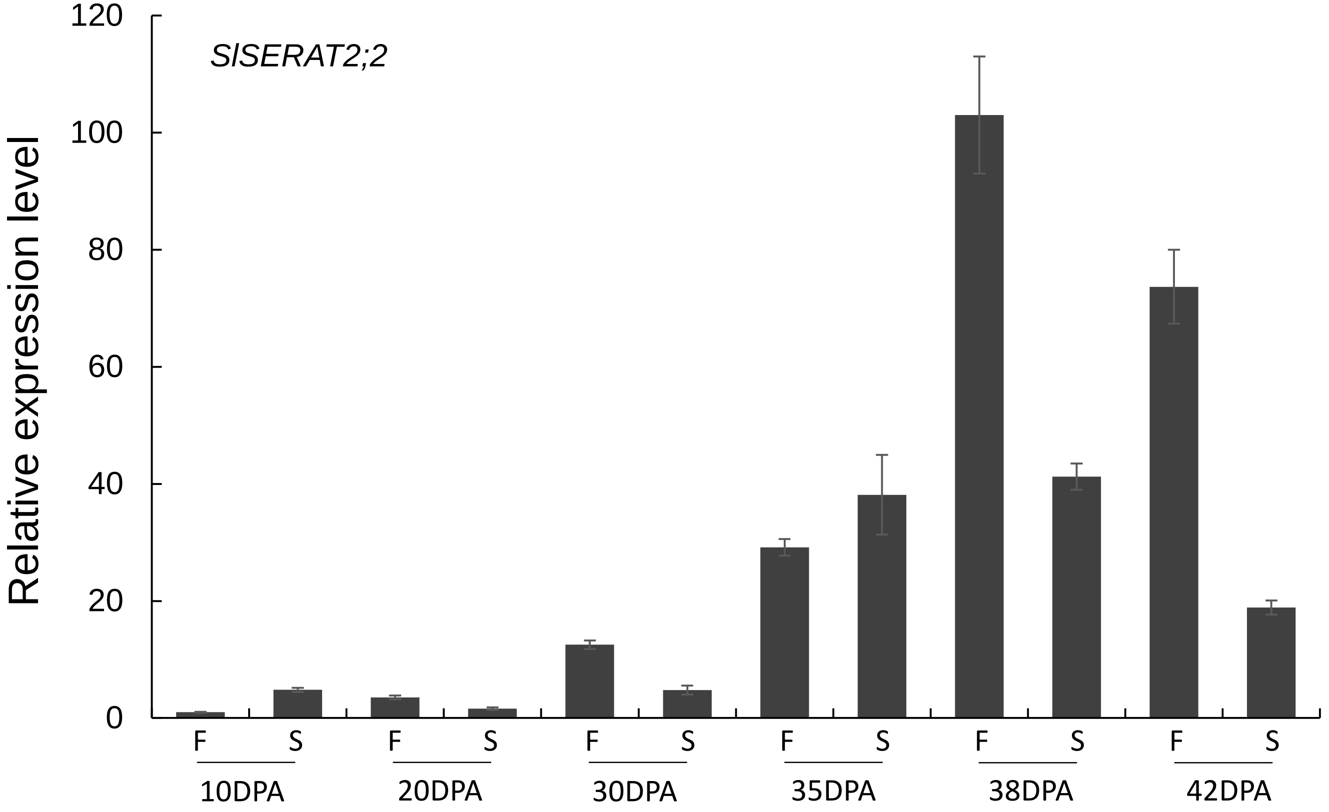

Fig. S5 The expression level changes of the *SlSERAT2;2* gene in different parts of the fruit during fruit development process. F, flesh; S, seeds; DPA, days after anthesis.

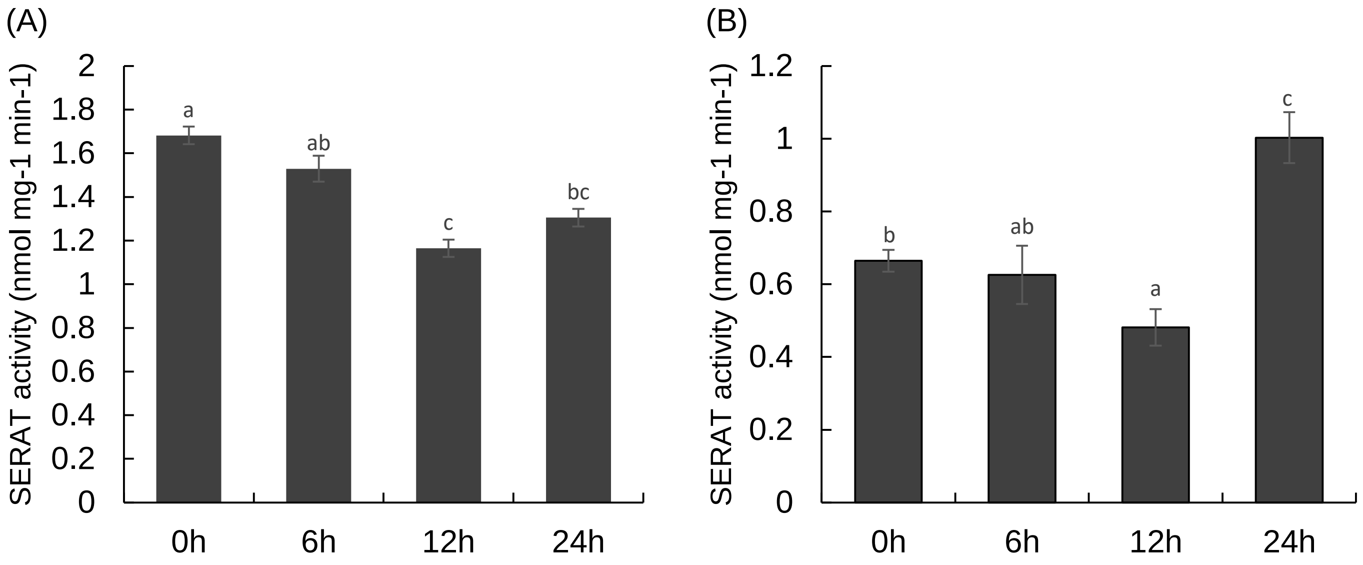

Figure S6 The changes of the total (A) and chloroplast (B) SERAT activity under salt stress

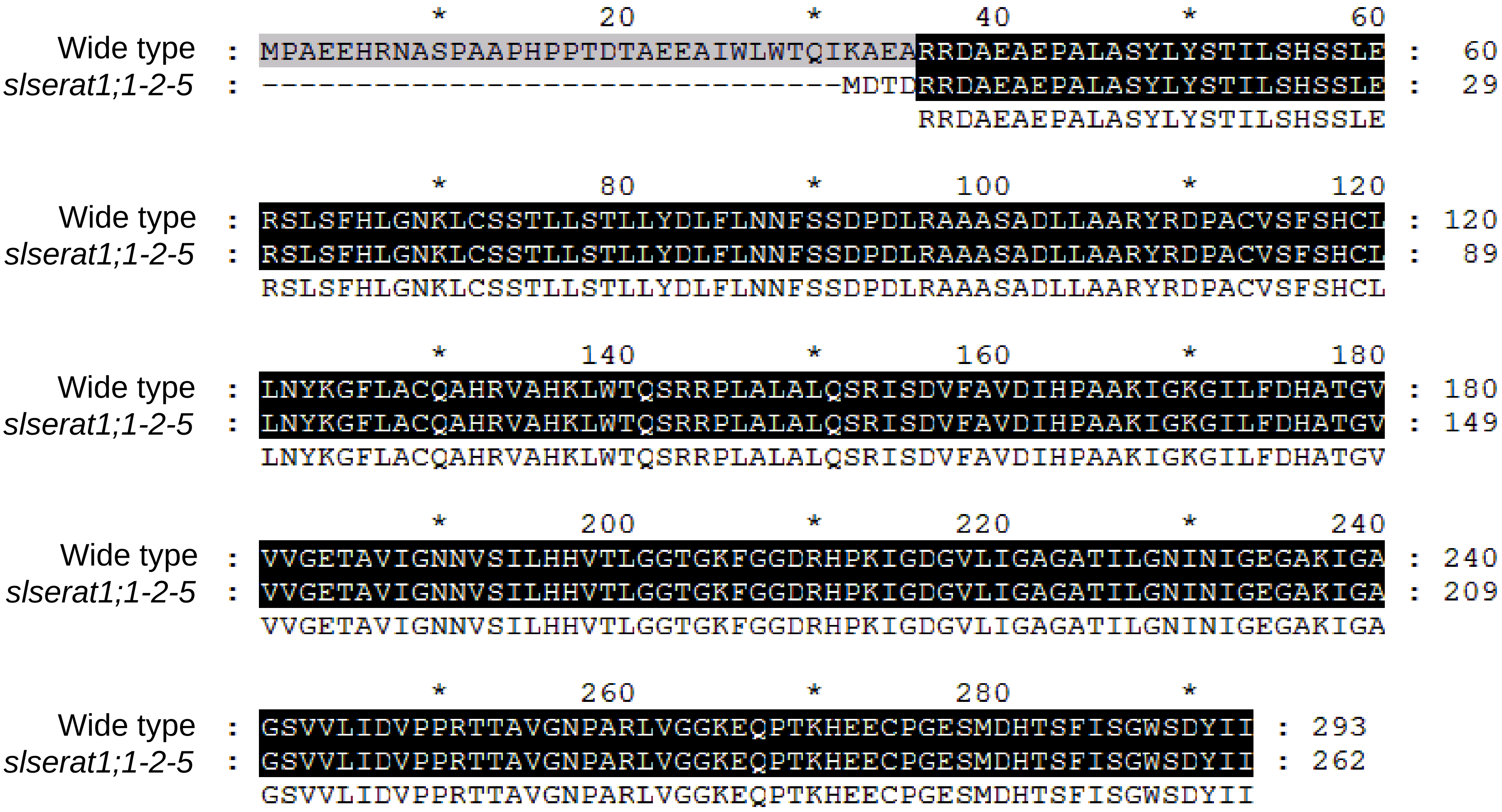

Fig. S7 The mutated protein sequence of SlSERAT1;1 in *slserat1;1-2-5*.

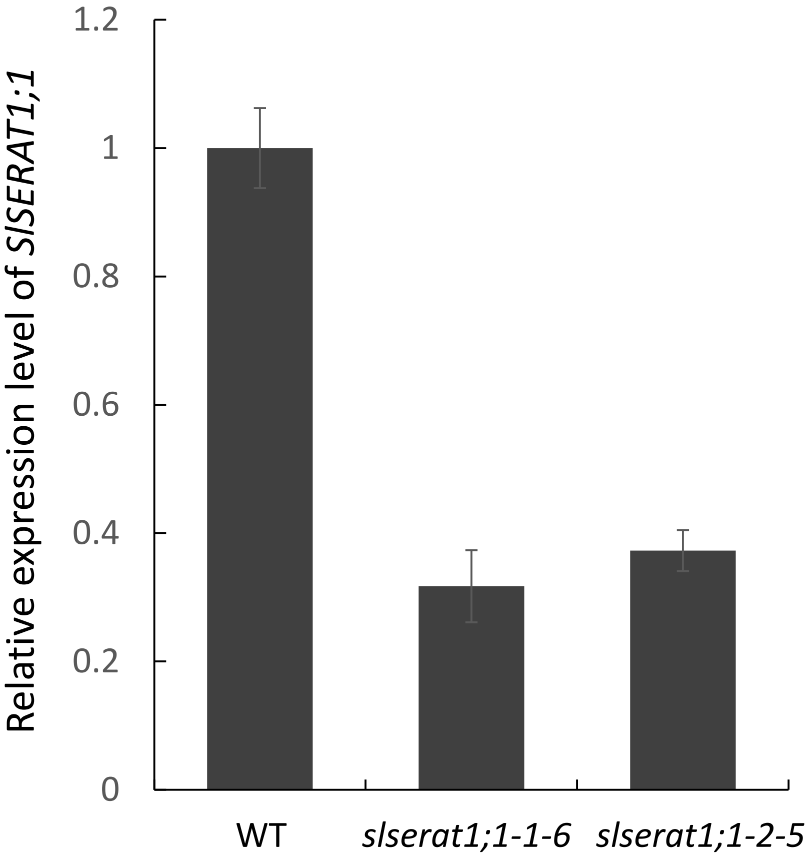

Fig. S8 The expression of *SlSERAT1;1* was downregulated in the *slserat1;1* mutants

Table S1 Primers used in this work

Assays	Destination products	Primer name	Primer sequence
Prokaryotic expression, over-expression	pCold-SlSERAT1;1 /pCAMBIA2300-N-FLAG-SlSERAT1;1	SlSERAT1;1-BamHI-F	CGCGGATCCATGCCAGCCGAAGAACACCG
		SlSERAT1;1-SalI-R	GACGTCGACTCAGATGATGTAATCAGACC
	pCold-SlSERAT2;1	SlSERAT2;1-XhoI-F	CCGCTCGAGATGTCCACTAATTTACTTGG
		SlSERAT2;1-SalI-R	GACGTCGACCTAAATTACATAATCAGAC
	pCold-SlSERAT2;2	SlSERAT2;2-BamHI-F	CGCGGATCCATGTACTCGAGTTTTATCGG
		SlSERAT2;2-SalI-R	GACGTCGACTTATATTACATAATCAGACC
	pCold-SlSERAT3;1	SlSERAT3;1-BamHI-F	CGCGGATCCATGGCTTGTTCTTGTTTGAG
		SlSERAT3;1-SalI-R	GACGTCGACTCATGTTGAGCCATCGTCC
	pET28a-SlOAS6	SlOAS6-BamHI-F	GCTGGATCCATGGCGGGGGAAAAGACTGG
		SlOAS6-SalI-R	GACGTCGACTCAAGGCTCCACAGTCATGT
Subcellular localiztion	p1305-SlSERAT1;1	SlSERAT1;1-XbaI-F	TGCTCTAGAATGCCAGCCGAAGAACACCG
		SlSERAT1;1-BamHI-R	CGCGGATCCGATGATGTAATCAGACCATC
	p1305-SlSERAT2;1	SlSERAT2;1-SpeI-F	CGGACTAGTATGTCCACTAATTTACTTGG
		SlSERAT2;1-XbaI-R	TGCTCTAGAAATTACATAATCAGACCAAT
	p1305-SlSERAT2;2	SlSERAT2;2-XbaI-F	TGCTCTAGAATGTACTCGAGTTTTATCGG
		SlSERAT2;2-BamHI-R	CGCGGATCCTATTACATAATCAGACCACT
	p1305-SlSERAT3;1	SlSERAT3;1-XbaI-F	TGCTCTAGAATGGCTTGTTCTTGTTTGAG
		SlSERAT3;1-BamHI-R	CGCGGATCCTGTTGAGCCATCGTCCTTTG
qRT-PCR		SlSERAT1;1-Q-F	ACGGACACGGCAGAAGAAG
		SlSERAT1;1-Q-R	ACAGGCTGGGTCACGGTAG
		SlSERAT2;1-Q-F	TAACCCAAACAAGTCTCAA
		SlSERAT2;1-Q-R	CATCAAATCTAGCCTCATC
		SlSERAT2;2-Q-F	ACTTTCTCGTGACCCAAAC
		SlSERAT2;2-Q-R	CAGCCATAACTCATCTCCC
		SlSERAT3;1-Q-F	ACAAATAATCGGACCCAA
		SlSERAT3;1-Q-R	GTAGAAGCGGTGTTAGCG
		SAND-F	TTGCTTGGAGGAACAGACG
		SAND-R	GCAAACAGAACCCCTGAATC
		ACTIN-F	GGTTTTGCTGGGGATGATGC
		ACTIN-R	CATGGCTGGACATTGAATGTCTC
Gene editing	pKI1.1R-SlSERAT1;1-1	SlSERAT1;1-Cr-F1	ATTGAGAACACCGTAACGCCTCGC
		SlSERAT1;1-Cr-R1	AAACGCGAGGCGTTACGGTGTTCT
	pKI1.1R-SlSERAT1;1-2	SlSERAT1;1-Cr-F2	ATTGCACAGATCAAAGCAGAAGCT
		SlSERAT1;1-Cr-R2	AAACAGCTTCTGCTTTGATCTGTG
Tomato mutants analysis		SlSERAT1;1-test-F	GACTCCGCCATCAAAACG
		SlSERAT1;1-test-R	ACAGGCTGGGTCACGGTAG
